# Selecting Some Variables to Update-Based Algorithm for Solving Optimization Problems

**DOI:** 10.3390/s22051795

**Published:** 2022-02-24

**Authors:** Mohammad Dehghani, Pavel Trojovský

**Affiliations:** Department of Mathematics, Faculty of Science, University of Hradec Králové, 500 03 Hradec Kralove, Czech Republic; mohammad.dehghani@uhk.cz

**Keywords:** stochastic methods, optimization, selected variables, optimization problem, population-based algorithm, population updating

## Abstract

With the advancement of science and technology, new complex optimization problems have emerged, and the achievement of optimal solutions has become increasingly important. Many of these problems have features and difficulties such as non-convex, nonlinear, discrete search space, and a non-differentiable objective function. Achieving the optimal solution to such problems has become a major challenge. To address this challenge and provide a solution to deal with the complexities and difficulties of optimization applications, a new stochastic-based optimization algorithm is proposed in this study. Optimization algorithms are a type of stochastic approach for addressing optimization issues that use random scanning of the search space to produce quasi-optimal answers. The Selecting Some Variables to Update-Based Algorithm (SSVUBA) is a new optimization algorithm developed in this study to handle optimization issues in various fields. The suggested algorithm’s key principles are to make better use of the information provided by different members of the population and to adjust the number of variables used to update the algorithm population during the iterations of the algorithm. The theory of the proposed SSVUBA is described, and then its mathematical model is offered for use in solving optimization issues. Fifty-three objective functions, including unimodal, multimodal, and CEC 2017 test functions, are utilized to assess the ability and usefulness of the proposed SSVUBA in addressing optimization issues. SSVUBA’s performance in optimizing real-world applications is evaluated on four engineering design issues. Furthermore, the performance of SSVUBA in optimization was compared to the performance of eight well-known algorithms to further evaluate its quality. The simulation results reveal that the proposed SSVUBA has a significant ability to handle various optimization issues and that it outperforms other competitor algorithms by giving appropriate quasi-optimal solutions that are closer to the global optima.

## 1. Introduction

The act of obtaining the optimal solution from multiple solutions under a given situation is known as optimization [1]. In designed problems in different sciences, items such as cost minimization, profit maximization, shortest length, maximum endurance, best structure, etc., are often raised, which require mathematical modeling of the problem based on the structure of an optimization problem and solving it with appropriate methods.

Mathematical methods of optimization are introduced according to the type of problem modeling, such as linear or nonlinear, constrained or non-constrained, continuous or linear programming, or nonlinear programming. Despite their good performance, these methods also have obstacles and disadvantages. These methods generally find the local optimal, especially if the initial guess is close to a local optimal. In addition, each of these methods assumes assumptions about the problem, which may not be true. These assumptions include derivability, convexity, and coherence. In addition to these disadvantages, the computation time of these methods in a group of optimization problems called nondeterministic polynomial-hard increases exponentially as the dimensions of the problem increase [2].

To overcome these challenges, a special class of optimization methods called stochastic-based optimization algorithms were developed. Because these algorithms rely on probabilistic and random search decisions and principles in many search steps of the optimal solution, these algorithms are called stochastic methods [3].

To find the best answer, optimization algorithms rely on a similar technique. The search procedure in most of these algorithms begins by generating a number of random answers within the allowable range of decision variables. This set of solutions in each of the algorithms has names such as population, colony, group, and so on. Moreover, each solution is assigned names such as chromosomes, ants, particles, and so on. The existing answers are then enhanced in various ways in an iterative process, and this action proceeds until the stop condition is achieved [4].

The global optimum is the fundamental answer to an optimization issue. However, optimization algorithms as stochastic methods are not necessarily able to supply the global optimal answer. Hence, the solution obtained from an optimization algorithm for an optimization problem is called quasi-optimal [5]. The criterion of goodness of a quasi-optimal solution depends on how close it is to the global optimal. As a result, when comparing the effectiveness of several optimization algorithms in addressing a problem, the method that produces a quasi-optimal solution that is closer to the global ideal optimal is preferable. This issue, as well as the goal to attain better quasi-optimal solutions, has prompted academics to extensive efforts and research to develop a variety of optimization algorithms that can provide solutions that are closer to the global optimal for optimization issues. Stochastic-based optimization algorithms have wide applications in optimization challenges in various sciences such as sensor networks [6], image processing [7], data mining [8], feature selection [9], clustering [10], engineering [11], the internet of things [12], and so on.

Is there still a need to develop new optimization algorithms despite the optimization algorithms that have been established so far? This is a key question that emerges in the research of optimization algorithms. The notion of the No Free Lunch (NFL) theorem has the answer to this question [13]. According to the NFL theorem, an optimization method that is effective in optimizing a group of optimization issues does not ensure that it will be useful in solving other optimization problems. As a result, it is impossible to say that one method is the best optimizer for all optimization problems. The NFL theorem motivates academics to create novel optimization algorithms to tackle optimization issues more efficiently.

The authors of this paper have developed several optimization algorithms in their previous works, such as the Pelican Optimization Algorithm (POA) [14] and Teamwork Optimization Algorithm (TOA) [15]. The common denominator of all optimization algorithms (both in the works of the authors of this article and the works of other researchers) can be considered the use of a random scan of the problem search space, random operators, no need for derivation process, easy implementation, simple concepts, and practicality in optimization challenges. The optimization process in population-based optimization algorithms starts with a random initial population. Then, in an iteration-based process, according to the algorithm steps, the position of the algorithm population in the search space is updated until the implementation is completed. The most important difference between optimization algorithms is in the same process of updating members of the algorithm population from one iteration to another. In POA, the algorithm population update process is based on simulating the strategies of pelicans while hunting. In TOA, modeling the activities and interactions of individuals in a group by presenting teamwork to achieve the team goal is the main idea in updating the population.

The novelty of this paper is in the development and design of a new optimization method named Selecting Some Variables to Update-Based Algorithm (SSVUBA) to address the optimization challenges and applications in various sciences. The main contributions of this paper are described as follows:

1. A new stochastic-based approach called Selecting Some Variables to Update-Based Algorithm (SSVUBA) used in optimization issues is introduced.
2. The fundamental idea behind the proposed method is to change the number of selected variables to update the algorithm population throughout iterations, as well as to use more information from diverse members of the population to prevent the algorithm from relying on one or several specific members.
3. SSVUBA theory and steps are described and its mathematical model is presented.
4. On a set of fifty-three standard objective functions of various unimodal, multimodal types, and CEC 2017, SSVUBA’s capacity to optimize is examined.
5. The proposed algorithm is implemented in four engineering design problems to analyze SSVUBA’s ability to solve real-world applications,
6. SSVUBA’s performance is compared to the performance of eight well-known algorithms to better understand its potential to optimize.

The following is the rest of the paper: A study of optimization methods is provided in Section 2. The proposed SSVUBA is introduced in Section 3. Simulation investigations are presented in Section 4. A discussion is provided in Section 5. The performance of SSVUBA in optimizing real-world applications is evaluated in Section 6. Section 7 contains the conclusions and recommendations for future research.

## 2. Background

Optimization algorithms are usually developed based on the simulation of various ideas in nature, physics, genetics and evolution, games, and any type of process that can be modeled as an optimizer.

One of the first and most prominent meta-heuristic algorithms is the Genetic Algorithm (GA), which is based on the theory of evolution. The main operator of this algorithm is a crossover that combines different members of the population together. However, the mutation operator is also useful for preventing premature convergence and falling into the local optimal trap. The smart part of this method is the selection stage, which in each stage, transmits better solutions to the next generation [16]. Ant Colony Optimization (ACO) is designed based on the inspiration of ants’ group behavior in food discovery. Ants release pheromones along the way to food. The presence of more pheromones in a path indicates the presence of a rich food source near that path. By modeling the process of pheromone release, pheromone tracking, and its evaporation with sunlight, the ACO is completed [17]. Particle Swarm Optimization (PSO) is one of the most established swarm-based algorithms, which is inspired by the social behavior of different biological species in their group life, such as birds and fish. This algorithm mimics the interaction between members to share information. Every particle is affected by its best situation and the best situation of the whole swarm, but it must move randomly [18]. The Simulated Annealing (SA) algorithm is a physics-based stochastic search method for optimization that relies on the simulation of the gradual heating and cooling process of metals called annealing. The purpose of annealing metals is to achieve a minimum energy and a suitable crystalline structure. In SA, this idea has been applied for optimization and search [19]. The Firefly Algorithm (FA) is based on the natural behavior of fireflies that live together in large clusters. FA simulates the activity of a group of fireflies by assigning a value to each firefly’s position as a model for the quantity of firefly pigments and then updating the fireflies’ location in subsequent iterations. The two main stages of FA in each iteration are the pigment update phase and the motion phase. Fireflies move toward other fireflies with more pigments in their neighborhood. In this way, during successive repetitions, the proposed solutions tend towards a better solution [20]. The Teaching–Learning Based Optimization (TLBO) method is based on simulating a teacher’s impact on the output of students in a classroom. TLBO is built on two fundamental modalities of teaching and learning: (1) Teacher phase in which knowledge is exchanged between the teacher and learners and (2) Learner phase in which knowledge is exchanged between learners and they learn from each other [21]. The Harmony Search (HS) method is one of the simplest optimization algorithms, and it is based on the simultaneous playing of a musical orchestra in the search for the best solution to optimization problems. To put it another way, the design of this algorithm is based on the idea that finding an optimal solution to a complicated issue is similar to the act of performing music [22]. The Artificial Fish Swarm Algorithm (AFSA) is one of the collective intelligence algorithms that is derived from the social behaviors of fish in nature and works based on random search and behaviorism. In the underwater world, fish can find areas that have more food, which is achieved by individual or group search of fish. According to this feature, AFSA is presented with the behaviors of free movement, food search, group movement, and tracking, by which the problem space is searched [23]. Gray Wolf Optimization (GWO) is a nature-inspired optimization technique based on the behavior of a wolf species known as the gray wolf. To mimic the leadership structure, four sorts of gray wolves designated Alpha, Beta, Delta, and Omega are employed in this program. Moreover, three basic hunting stages have been modeled for solution updating: prey search, prey siege, and prey attack [24]. The Gravitational Search Method (GSA) is a physics-based approach that is built on simulating the law of gravitational pull between masses at different distances from each other. In GSA, the process of updating population members is based on calculating the gravitational force between masses and then implementing Newton’s laws of motion [25]. The Whale Optimization Algorithm (WOA) is a nature-based optimizer that depicts humpback whale social behavior. In WOA, the search agents’ position is updated in each iteration using three operators: prey siege, bubble-net attack method (exploitation stage), and prey search (exploration stage) [26]. The Marine Predators Algorithm (MPA) is a bio-inspired optimizer that is inspired by the marine predators’ movement strategies when trapping prey in the oceans. In MPA, population members are updated based on three different strategies in each iteration: (i) prey speed is faster than predator speed, (ii) prey and predator speeds are almost equal, and (iii) predator speed is faster than prey speed [27]. The Tunicate Swarm Algorithm (TSA) is a nature-inspired based optimizer that is built on simulations of swarm behavior and jet propulsion of the tunicate when finding a food source. In TSA, the jet propulsion behavior is modelled based on three principles: (i) preventing clashes between search agents, (ii) movement in the best neighbor’s direction, and (iii) converging towards the best search agent [28]. The Quantum-based Avian Navigation Algorithm (QANA) is an optimizer that is formed based on the simulation of the extraordinary precision navigation of migratory birds during long-distance aerial paths [29]. The Conscious neighborhood-based Crow Search Algorithm (CCSA) is a bio-inspired method that is introduced by imitation of the natural behaviors of crow and employs three search strategies: wandering around-based search, non-neighborhood-based global search, and neighborhood-based local search [30]. The Black Widow Optimization Algorithm (BWO) is a swarm-based technique that is proposed based on the mating behavior of black widow spiders in nature [31]. The Red Fox Optimization Algorithm (RFO) is a bio-inspired method that is produced based on natural behaviors of red fox habits including hunting, searching for food, and escaping mechanisms [32]. The Artificial Hummingbird Algorithm (AHA) is a swarm intelligence optimizer that is developed based on the simulation of the intelligent foraging behaviors and special flight abilities of hummingbirds in nature [33]. The Reptile Search Algorithm (RSA) is a nature-inspired optimizer that is formed based on the hunting behaviors of crocodiles. Two crocodile strategies, encircling and cooperation in hunting, have been employed in RSA design [34]. The Honey Badger Algorithm (HBA) is a bio-inspired technique that is developed based on the intelligent foraging behavior of honey badger. In the design of HBA, in addition to the search behavior of honey badgers, their honey-finding and digging strategies are also employed and modeled [35]. The Starling Murmuration Optimizer (SMO) is a bio-inspired algorithm that is formed based on the imitation of the starlings’ behaviors during their stunning murmuration. SMO uses three strategies, whirling, separating, and diving, to achieve solutions to optimization problems [36].

## 3. Selecting Some Variables to Update-Based Algorithm (SSVUBA)

In this section, the theory and all stages of the Selecting Some Variables to Update-Based Algorithm (SSVUBA) are described, and then its mathematical model is presented for application in tackling optimization issues.

### 3.1. Mathmatical Model of SSVUBA

SSVUBA is a population-based stochastic algorithm. Each optimization issue has a search space with the same number of axes as the problem’s variables. According to its position in the search space, each member of the population assigns values to these axes. As a result, each member of the population in the SSVUBA is a proposed solution to the optimization issue. Each member of the population can be mathematically described as a vector, each component of which represents the value of one of the problem variables. As a result, the population members of the proposed SSVUBA can be modeled using a matrix termed the population matrix, as shown in Equation (1).

(1) $$X={\left[\begin{array}{c} X_{1}\\ \vdots \\ X_{i}\\ \vdots \\ X_{N} \end{array}\right]}_{N\times m}={\left[\begin{array}{ccccc} x_{1,1} & \cdots & x_{1,d} & \cdots & x_{1,m}\\ \vdots & \ddots & \vdots & ⋰ & \vdots \\ x_{i,1} & \cdots & x_{i,d} & \cdots & x_{i,m}\\ \vdots & ⋰ & \vdots & \ddots & \vdots \\ x_{N,1} & \cdots & x_{N,d} & \cdots & x_{N,m} \end{array}\right]}_{N\times m},$$

where X is the SSVUBA’s population matrix, $X_{i}$ is the *i*th member, $x_{i,d}$ is the value of the *d*th problem variable generated by the *i*th member, N is the number of population members, and m is the number of problem variables.

The objective function of the problem can be assessed using the theory that each member of the population provides values for the problem variables. As a result, the values derived for the objective function based on the evaluation of different members of the population can be described employing a vector according to Equation (2).

(2) $$F={\left[\begin{array}{c} F_{1}\\ \vdots \\ F_{i}\\ \vdots \\ F_{N} \end{array}\right]}_{N\times 1}={\left[\begin{array}{c} F\left(X_{1}\right)\\ \vdots \\ F\left(X_{i}\right)\\ \vdots \\ F\left(X_{N}\right) \end{array}\right]}_{N\times 1},$$

where F denotes the objective function vector and $F_{i}$ represents the objective function value obtained from the *i*th population member’s evaluation.

The process of updating population members in the proposed SSVUBA adheres to two principles.

The first principle is that some members of the population may be in a situation where if only the values of some variables change, they will be in a better position instead of changing all of the variables. Therefore, in the proposed SSVUBA, the number of variables selected for the update process is set in each iteration. In this way, in the initial repetitions, the number is set to the maximum and at the end of the repetitions to the minimum number of variables. This principle is mathematically simulated using an index based on Equation (3).

(3) $$I_{v}=\mathrm{round}\left(\left(1-\frac{t}{T}\right)\cdot m\right),$$

where $I_{v}$ denotes the number of selected variables for the update process, T is the maximum number of iterations, and t is the repetition counter.

The second principle is to prevent the algorithm population update process from relying on specific members. Relying on algorithm updates to specific members of the population might cause the algorithm to converge towards the local optimum and prevent accurate scanning of the search space to attain the global optimum. The process of updating population members has been modeled using Equations (4)–(6) according to the two principles expressed. To update each member of the population, another member of the population is randomly selected. If the selected member has a better value for the objective function, the first formula in Equation (4) is used. Otherwise, the second formula is used.

(4) $$X_{i}^{new}:\;x_{i,k_{j}\;}^{new}=\{\begin{array}{c} x_{i,k_{j}}+r\cdot \left(x_{s,k_{j}}-I\cdot x_{i,k_{j}}\right),\;\;\;F_{s}<F_{i},\\ x_{i,k_{j}}+r\cdot \left(x_{i,k_{j}}-I\cdot x_{s,k_{j}}\right),\;\;\;else, \end{array}$$

(5) I=round(1+r),

(6) $$X_{i}=\{\begin{array}{cc} X_{i}^{new}, & F_{i}^{new}<F_{i},\\ X_{i}, & else, \end{array}$$

where $X_{i}^{new},\;i=1,2,\;\ldots ,\;N,\;$is the new status of the *i*th member,$\;x_{i,k_{j}\;}^{new},$ $j=1,2,\ldots ,I_{v},$ $k_{j}\;$is a random element from the set {1,2, …,m} is the *k_j_*th dimension of the *i*th member, $F_{i}^{new}$ is the objective function value of the *i*th population member in new status, r is a random number in interval [0, 1], $x_{s,k_{j}}$ is the selected member for guiding the *i*th member in the *k_j_*th dimension, and $F_{s}$ is the its objective function value.

### 3.2. Repetition Process of SSVUBA

**Figure 1:**
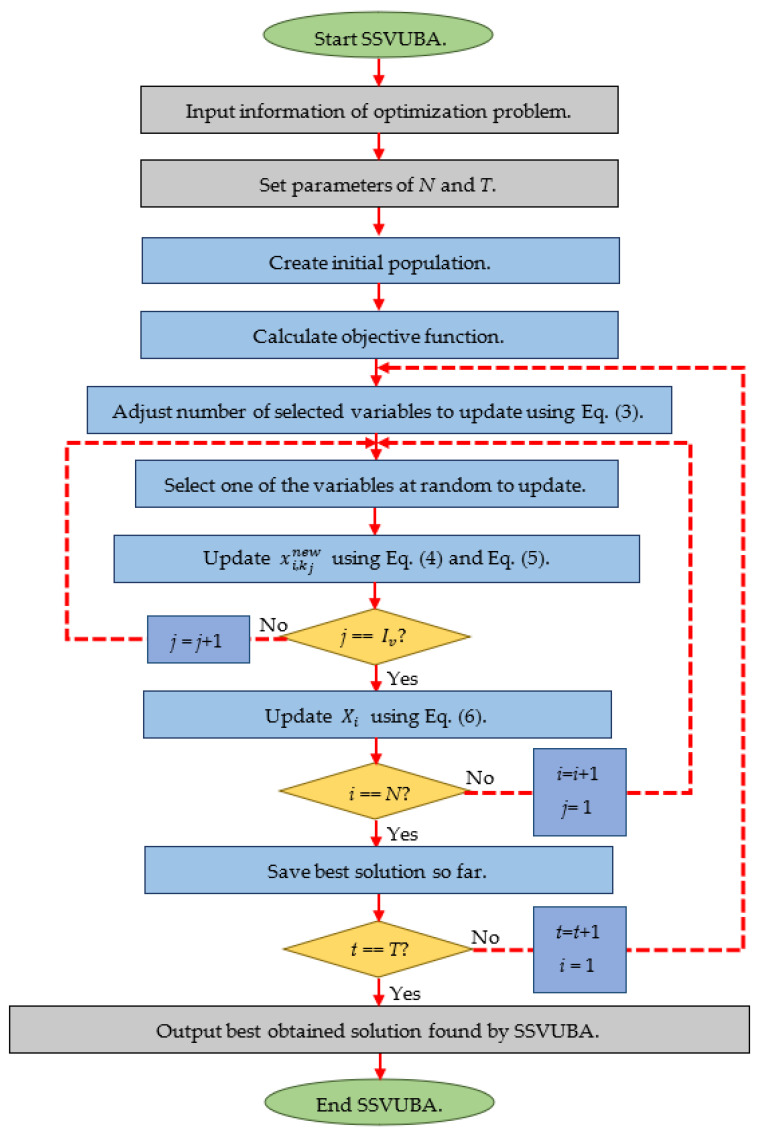
Flowchart of SSVUBA.

After all members of the population have been updated, the SSVUBA algorithm goes on to the next iteration. In the new iteration, index $I_{v}$ is adjusted using Equation (3), and then population members are updated based on Equations (4)–(6). This process repeats until the algorithm is completed. The best quasi-optimal solution found by the algorithm during execution is offered as the answer to the problem after the complete implementation of SSVUBA for the specified optimization problem. Figure 1 depicts the flowchart of the SSVUBA’s various steps, while Algorithm 1 presents its pseudocode.

### 3.3. Computational Complexity of SSVUBA

In this subsection, the computational complexity of SSVUBA is presented. In this regard, time and space complexities are discussed.

#### 3.3.1. Time Complexity

SSVUBA preparation and initialization require O(N·m) time where N is the number of SVVUBA population members and m is the number of problem variables. In each iteration of the algorithm, population members are updated, which requires $O\left(T\cdot N\cdot I_{v}\right)\;$time where T is the maximum number of iteations and $I_{v}$ is the number of selected variables for the update process. Accordingly, the total time computational complexity of SSVUBA is equal to $O(N\left(m+T\cdot I_{v}\right)$).

#### 3.3.2. Space Complexity

The space complexity of SSVUBA is equal to O(N·m), which is considered the maximum value of space pending its initialization procedure.
**Algorithm 1.** Pseudo-code of SSVUBA**Start SSVUBA.**1.     Input the optimization problem information: Decision variables, constraints, and objective function2.     Set the *T* and *N* parameters.3.     **For *t* = 1:*T***
4.
           $\mathrm{Adjust}\;\mathrm{number}\;\mathrm{of}\;\mathrm{selected}\;\mathrm{variables}\;\mathrm{to}\;\mathrm{update}\;(I_{v}$
$)\;\mathrm{using}\;\mathrm{Equation}\;(3).\;I_{v}\leftarrow \mathrm{round}\left(\left(1-\frac{t}{T}\right)\cdot m\right)$
5.
           **For i = 1:*N***
6.

                 **For j = 1:**
$\;I_{v}$
7.


                       Select a population member randomly to guide the *i*th population member.                        $X_{S}\leftarrow X\left(S,:\right),\;S\;randomly\;selected\;from\;\left\{1,2,\;\ldots ,\;N\right\}\;and\;S\neq i$, is the *S*th row of the population matrix. 8.


                       Select one of the variables at random to update. $x_{i,k_{j}},\;k_{j}\;randomly\;selected\;from\;\left\{1,2,\;\ldots ,m\right\}$.9.


                       Calculate I using Equation (5). I←round(1+r)
10.


                       **If**
$F_{s}<F_{i}$
11.



                          $\mathrm{Calculate}\;\mathrm{the}\;\mathrm{new}\;\mathrm{status}\;\mathrm{of}\;\mathrm{the}\;k_{j}\mathrm{th}\;\mathrm{dimension}\;\mathrm{using}\;\mathrm{Equation}\;(4).\;x_{i,k_{j}\;}^{new}\leftarrow x_{i,k_{j}}+r\cdot \left(x_{s,k_{j}}-I\cdot x_{i,k_{j}}\right)$
12.


                       else13.



                          $\mathrm{Calculate}\;\mathrm{the}\;\mathrm{new}\;\mathrm{status}\;\mathrm{of}\;\mathrm{the}\;k_{j}\mathrm{th}\;\mathrm{dimension}\;\mathrm{using}\;\mathrm{Equation}\;(4).\;x_{i,k_{j}\;}^{new}\leftarrow x_{i,k_{j}}+r\cdot \left(x_{i,k_{j}}-I\cdot x_{s,k_{j}}\right)$
14.


                       **end**
15.

                 **end**
16.

                 $\mathrm{Calculate}\;\mathrm{the}\;\mathrm{objective}\;\mathrm{function}\;\mathrm{based}\;\mathrm{on}\;X_{i}^{new}$
$.\;F_{i}^{new}\leftarrow F\left(X_{i}^{new}\right)$
17.

                 **If**
$F_{i}^{new}<F_{i}$
18.


                    $\mathrm{Update}\;\mathrm{the}\;i\mathrm{th}\;\mathrm{population}\;\mathrm{member}\;\mathrm{using}\;\mathrm{Equation}\;(6).\;X_{i}\leftarrow X_{i}^{new}$
19.

                 **else**
20.


                    $\mathrm{Update}\;\mathrm{the}\;i\mathrm{th}\;\mathrm{population}\;\mathrm{member}\;\mathrm{using}\;\mathrm{Equation}\;(6).\;X_{i}\leftarrow X_{i}$
21.

                 **end**
22.
           **end**
23.
           Save the best solution so far.24.     **end**
25.     Output the best obtained solution.**End SSVUBA.**








### 3.4. Visualization of the Movement of Population Members towards the Solution

In the SSVUBA approach, population members converge to the optimal area and solution in the search space under the exchange of information between each other and the algorithm steps. In this subsection, to provide the visualization of the members’ movement in the search space, the process of SSVUBA members’ access to the solution is intuitively shown. This visualization is presented in a two-dimensional space, with a population size equals 30 and 30 iterations in optimizing an objective function called the Sphere function; its mathematical model is as follows:

$$F\left(x_{1},x_{2}\right)=x_{1}^{2}+x_{2}^{2}$$

Subject to:

$$-10\leq x_{1},x_{2}\leq 10$$

**Figure 2:**
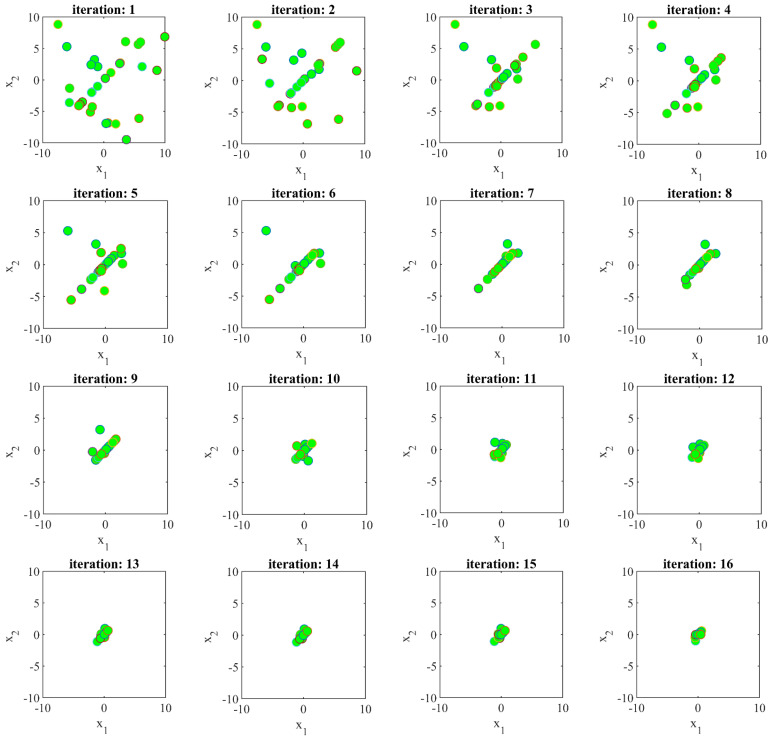

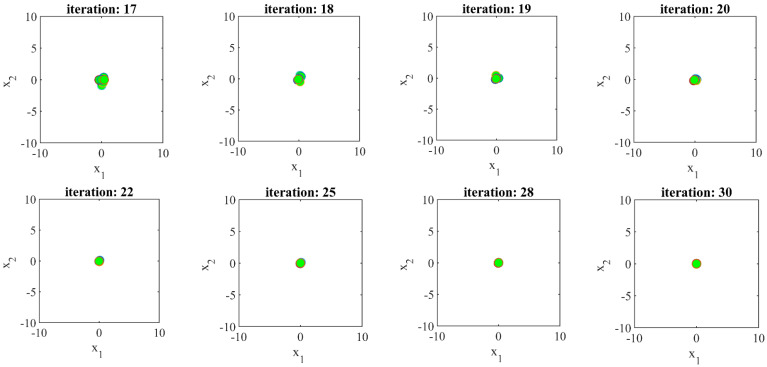
Visualization of the movement of SSVUBA members towards the solution in the search space.

Figure 2 shows the process of achieving SSVUBA towards the solution by optimizing the mentioned objective function. In this figure, the convergence of the population members towards the optimal solution of the variables (i.e., $x_{1}=x_{2}=0$) and the optimal value of the objective function (i.e., $F\left(x_{1},x_{2}\right)=0$) is well evident.

## 4. Simulation Studies and Results

In this section, simulation studies are presented to evaluate the performance of the SSVUBA in optimization and provide appropriate solutions for optimization problems. For this purpose, the SSVUBA is utilized for twenty-three standard objective functions of unimodal, high-dimensional multimodal, and fixed-dimensional multimodal types [37] (see their definitions in Appendix A). In addition to the twenty-three objective functions, SSVUBA performance has been tested in optimizing CEC 2017 test functions [38] (see their definitions in Appendix A). Furthermore, the optimization results achieved for the above objective functions using SSVUBA are compared to the performance of twelve optimization methods: PSO, TLBO, GWO, WOA, MPA, TSA, GSA, GA, RFO, RSA, AHA, and HBA to assess the further proposed approach. Numerous optimization algorithms have been developed so far. Comparing an algorithm with all existing algorithms, although possible, will yield a large amount of results. Therefore, twelve optimization algorithms have been used to compare the results. The reasons for choosing these algorithms are as follows: (i) Popular and widely used algorithms: GA and PSO. (ii) Algorithms that have been widely cited and employed in a variety of applications: GSA, TLBO, GWO, WOA. (iii) Algorithms that have been published recently and have received a lot of attention: RFO, TSA, MPA, RSA, AHA, HBA. The average of the best obtained solutions (avg), the standard deviation of the best obtained solutions (std), the best obtained candidate solution (bsf), and the median of obtained solutions (med) are used to present the optimization outcomes of objective functions. Table 1 shows the values utilized for the control parameters of the compared optimization techniques.

### 4.1. Assessment of F1 to F7 Unimodal Functions

Unimodal functions are the first category of objective functions that are considered for analyzing the performance of optimization methods. The optimization results of unimodal objective functions including F1 to F7 using SSVUBA and eight compared algorithms are reported in Table 2. The SSVUBA has been able to find the global optimal for the F6 function. Further, SSVUBA is the first best optimizer for the F1 to F5 and F7 functions. Analysis of the performance of optimization algorithms against the results of the proposed approach indicates that SSVUBA is able to provide quasi-optimal solutions closer to the global optimum and thus has a higher capability in optimizing unimodal functions than the compared algorithms.

### 4.2. Assessment of F8 to F13 High-Dimensional Multimodal Functions

High-dimensional multimodal functions are the second type of objective function employed to assess the performance of optimization techniques. Table 3 reveals the results of the implementation of the SSVUBA and eight compared algorithms for functions F8 to F13. For the F9 and F11 functions, SSVUBA was able to deliver the best global solution. Furthermore, for the F8, F10, F12, and F13 functions, SSVUBA was the superior optimizer. SSVUBA outperformed the other algorithms in solving high-dimensional multimodal issues by offering effective solutions for the F8 to F13 functions, according to the simulation findings.

### 4.3. Assessment of F14 to F23 Fixed-Dimensional Multimodal Functions

**Figure 3:**
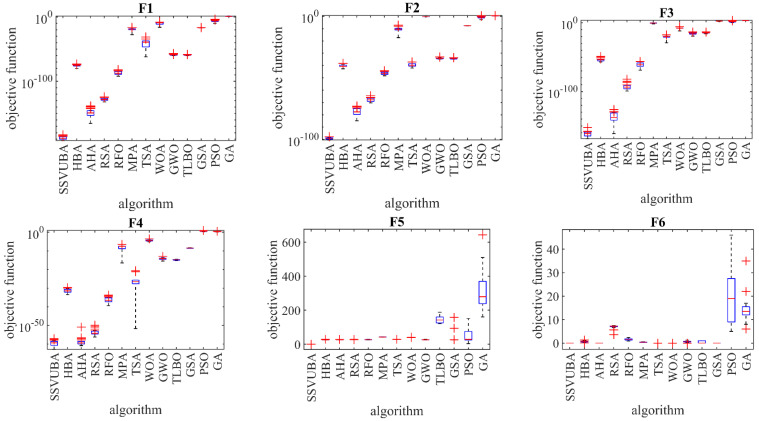

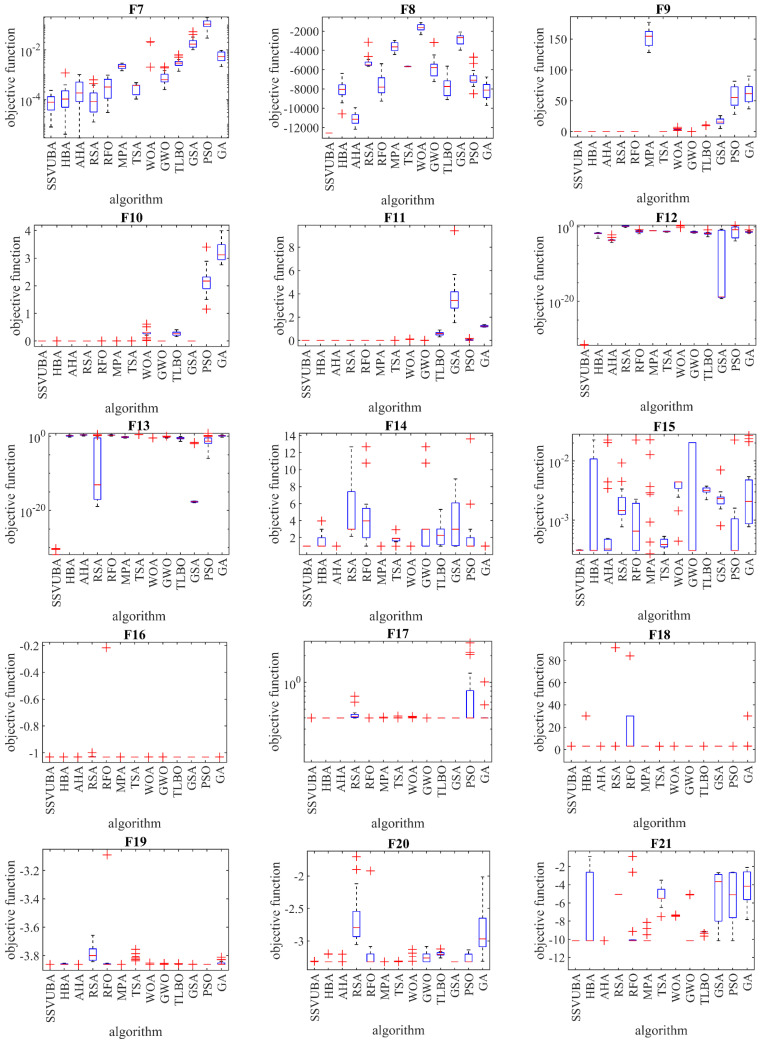

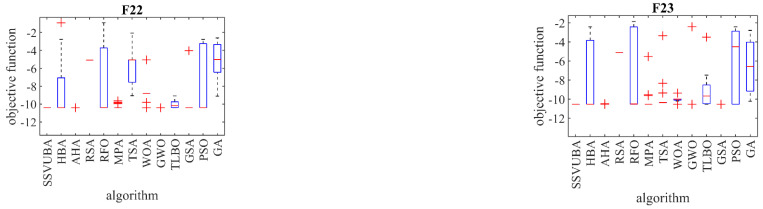
Boxplot displaying SSVUBA performance against compared algorithms in the F1 to F23 optimization.

Fixed-dimensional functions are the third type of objective function used to evaluate the efficiency of optimization techniques. Table 4 shows the optimization results for the F14 to F23 functions utilizing the SSVUBA and eight compared techniques. SSVUBA was able to deliver the global optimum for the F14 function. The SSVUBA was also the first best optimizer for the F15, F16, F21, and F22 functions. SSVUBA, in optimizing functions F17, F18, F19, F20, and F23, was able to converge to quasi-optimal solutions with smaller values of the standard deviation. By comparing the performance of optimization algorithms in solving the F14 to F23 functions, it is clear that SSVUBA provides superior and competitive results versus the compared algorithms. Figure 3 shows the performance of SSVUBA as well as eight competitor algorithms in the form of a boxplot.

### 4.4. Statistical Analysis

Use of the average of the obtained solutions, standard deviation, best candidate solution, and median of obtained solutions to analyze and compare the performance of optimization algorithms in solving optimization issues offers significant information about the quality and capabilities of optimization algorithms. However, it is possible that the superiority of one algorithm among several algorithms in solving optimization problems is random by even a low probability. Therefore, in this subsection, in order to statistically analyze the superiority of SSVUBA, the Wilcoxon sum rank test [39] is used. The Wilcoxon rank sum test is a nonparametric test to assess whether the distributions of results obtained between two separate methods for a dependent variable are systematically different from one another.

The Wilcoxon rank sum test was implemented for the optimization results obtained from the optimization algorithms. The results of this analysis are presented in Table 5. In the Wilcoxon rank sum test, a *p*-value indicates whether the superiority of one algorithm over another is significant. Therefore, the proposed SSVUBA in cases where the *p*-value is less than 5% has a statistically significant performance superior to the compared algorithm.

### 4.5. Sensitivity Analysis

The proposed SSVUBA is a population-based algorithm that is able to solve optimization problems in an iteration-based procedure. Therefore, the two parameters *N* and *T* affect the performance of SSVUBA in achieving the solution. As a result, the sensitivity analysis of the proposed SSVUBA to these two parameters is described in this subsection.

**Figure 4:**
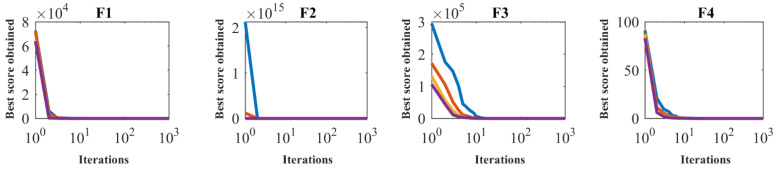

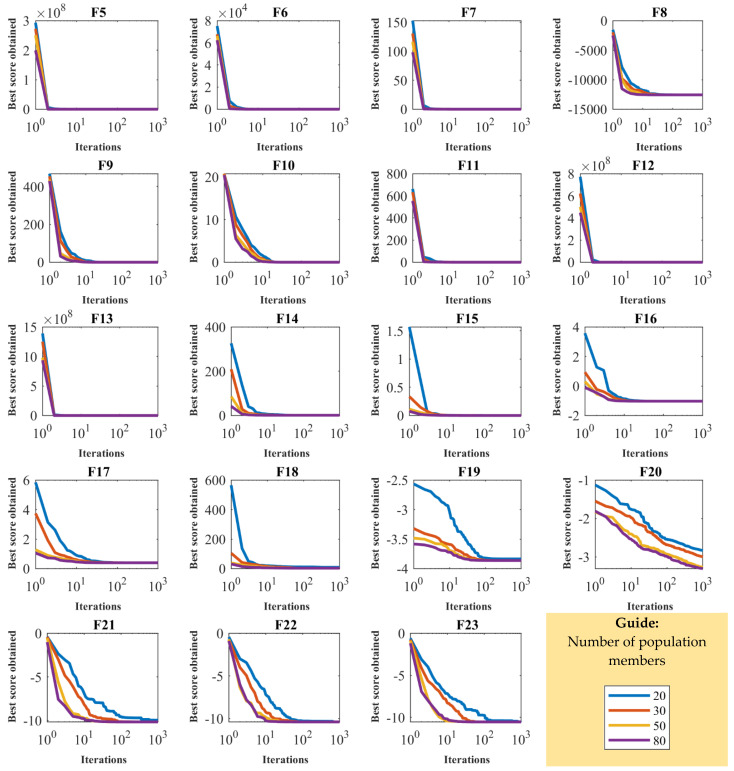
Sensitivity analysis of the SSVUBA for the number of population members.

SSVUBA has been applied to F1 to F23 functions in independent runs for different populations with 20, 30, 50, and 80 members to investigate the sensitivity of the proposed SSVUBA performance to the *N* parameter. Table 6 reveals the findings of SSVUBA’s sensitivity analysis to *N*. In addition, the convergence curves of the proposed SSVUBA to attain a quasi-optimal solution for different populations are plotted in Figure 4. The sensitivity analysis of the SSVUBA to the number of population members show that increasing the search agents in the search space leads to more accurate scanning of the search space and achieving more appropriate optimal solutions.

**Figure 5:**
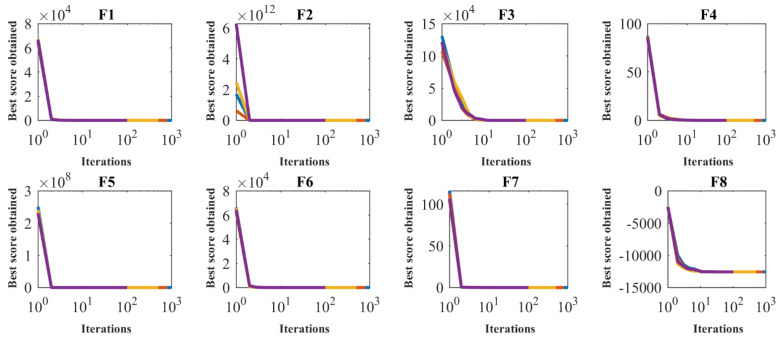

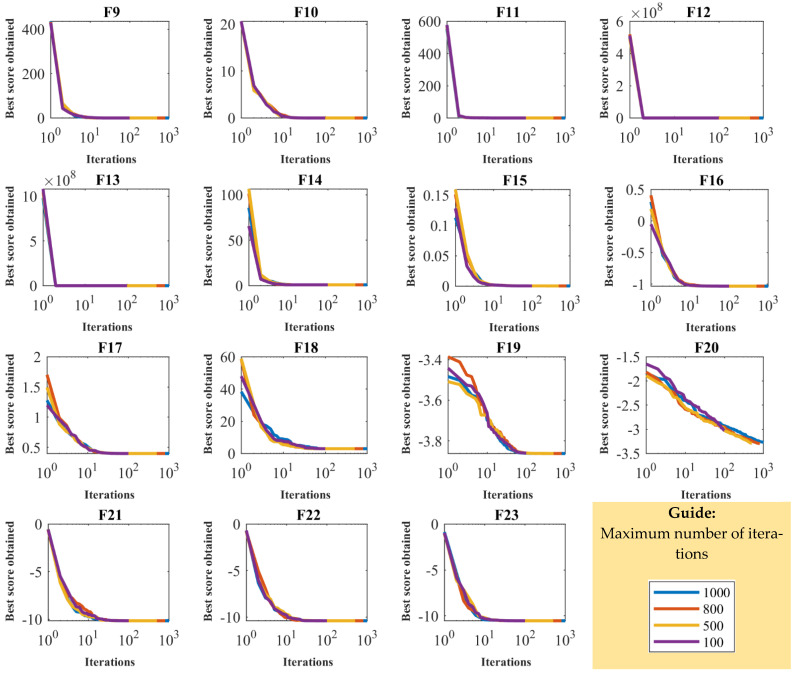
Sensitivity analysis of the SSVUBA for the maximum number of iterations.

The proposed approach is implemented in independent performances for the number of iterations 100, 500, 800, and 1000 in order to optimize the objective functions F1 to F23 with aim of the investigating the sensitivity of the performance of SSVUBA to parameter *T*. Table 7 shows the simulated results of this sensitivity study, and Figure 5 shows the convergence curves of the SSVUBA under the influence of this analysis. The results of the simulation and sensitivity analysis of the proposed algorithm to the parameter *T* illustrate that increasing the number of iterations of the algorithm provides more opportunity for the algorithm to converge towards optimal solutions. As a result, as the maximum number of iterations increases and the values of the objective functions decrease.

**Figure 6:**
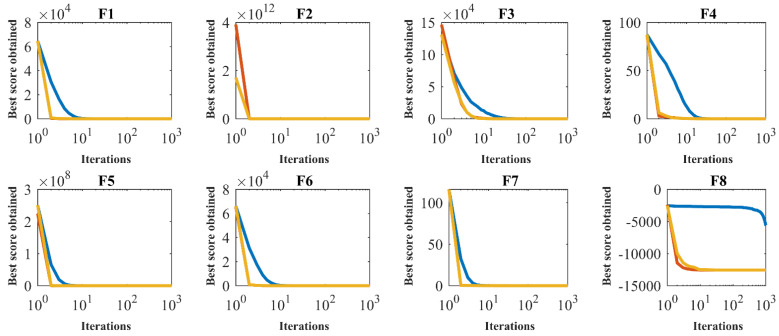

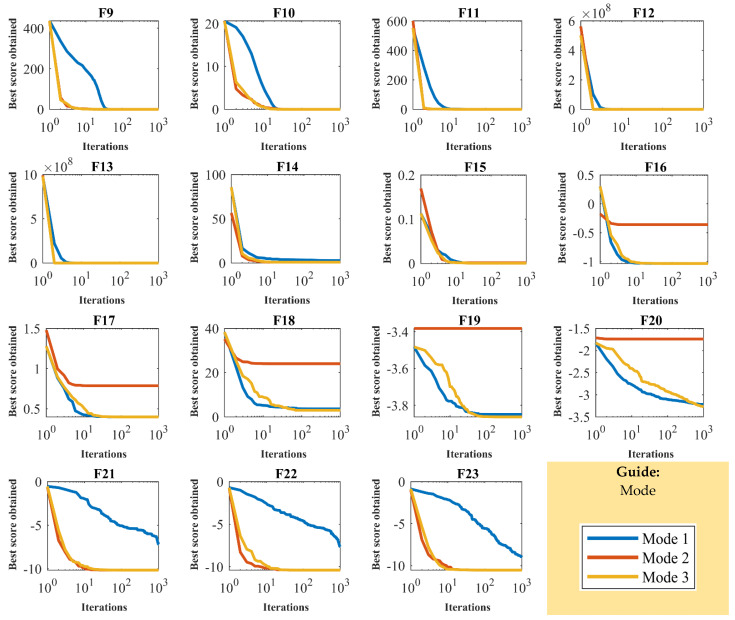
Sensitivity analysis of the SSVUBA to effectiveness of each case in Equation (4).

In addition to studying the analysis of SSVUBA sensitivity to the N and T parameters, each of the relationships used in Equation (4) also affects the performance of SSVUBA. Therefore, the effectiveness of all cases in Equation (4) is examined at this stage. In this regard, the proposed SSVUBA is implemented in three different modes for the objective functions F1 to F23. In the first case (mode 1), the first case of Equation (4), i.e., $x_{i,k_{j}}+r\cdot \left(x_{s,k_{j}}-I\cdot x_{i,k_{j}}\right)$ is used. In the second case (mode 2), the second case of Equation (4), i.e., $x_{i,k_{j}}+r\cdot \left(x_{i,k_{j}}-I\cdot x_{s,k_{j}}\right)$ is used. In the third case (mode 3), both cases introduced in Equation (4) are used simultaneously. The results of this analysis are shown in Table 8, and Figure 6 shows the SSVUBA convergence curves in the optimization of functions F1 to F23 in this study. What can be deduced from the simulation results is that applying the relationships in Equation (4) simultaneously has led to better and more efficient optimization results for the objective functions F1 to F23 compared to using each of the relationships separately.

### 4.6. Population Diversity Analysis

Population diversity has a significant impact on the success of the optimization process by optimization algorithms. Population diversity can improve the algorithm’s ability to search globally in the problem-solving space, thus preventing it from falling into the trap of local optimal solutions. In this regard, in this subsection, population diversity analysis of SSVUBA performance has been studied. To show the population diversity of SSVUBA in achieving the solution during the iterations of the algorithm, the $I_{C}$ index is used, which is calculated using Equations (7) and (8) [40].

(7) $$I_{C}=\sum_{j=1}^{m}\sum_{i=1}^{N}{\left(x_{i,j}-c_{j}\right)}^{2}$$

(8) $$c_{j}=\frac{1}{N}\sum_{i=1}^{N}x_{i,j}$$

Here, $I_{C}$ is the spreading of each population member from its centroid and $c_{j}$ is its centroid.

**Figure 7:**
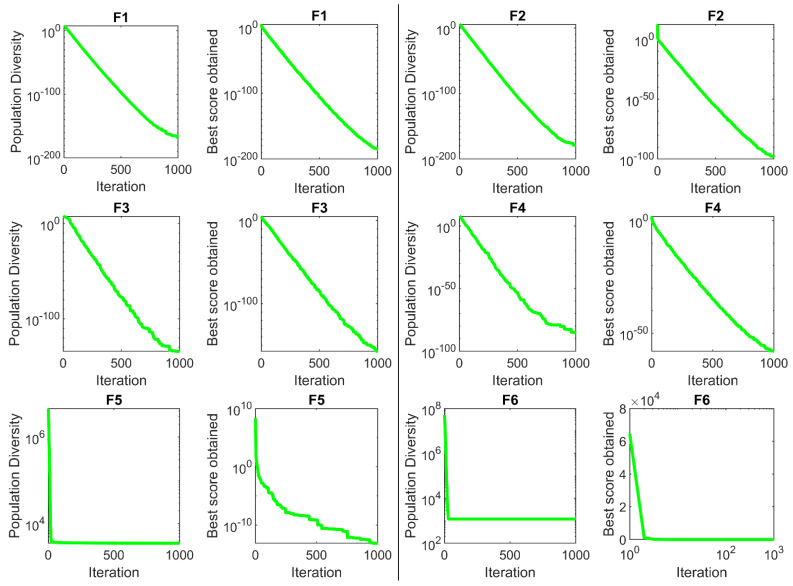

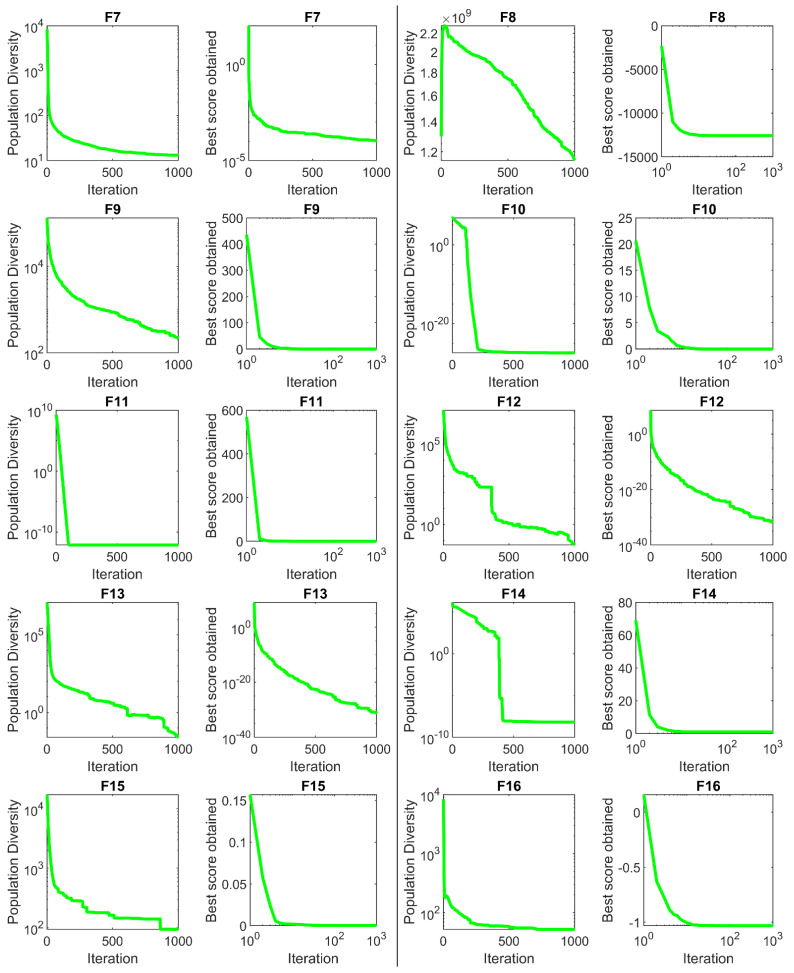

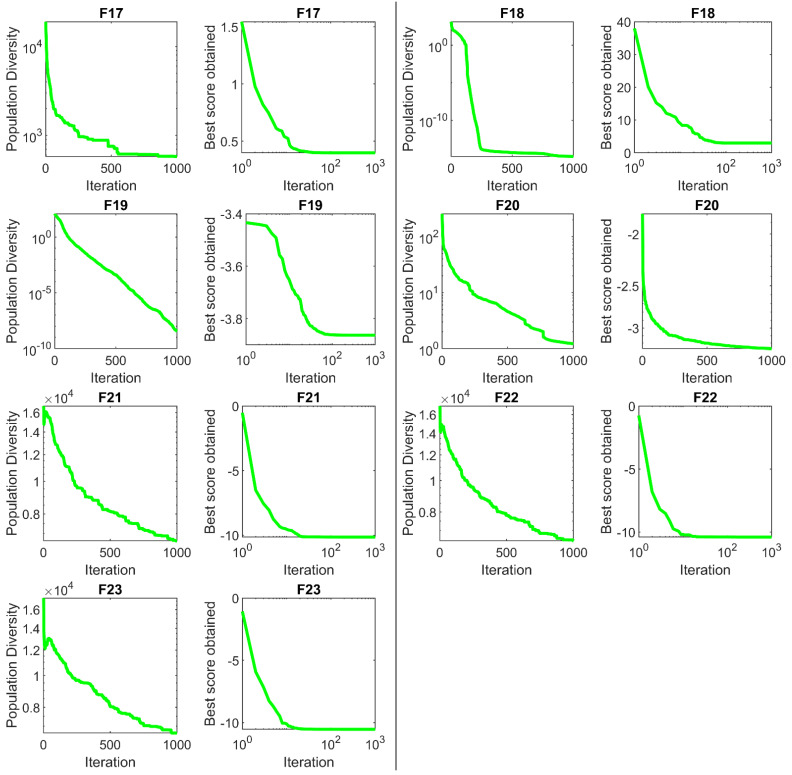
The population diversity and convergence curves of the SSVUBA.

The impact of population diversity on the optimization process given by SSVUBA in optimizing functions F1 to F23 is shown in Figure 7. In this figure, population diversity and SSVUBA convergence curves are presented for each of the objective functions. As can be seen from the simulation results, SSVUBA has a high population diversity in the process of optimizing most of the target functions. By optimizing functions F1, F2, F3, F4, F7, F8, F9, F12, F13, F15, F16, F17, F18, F19, F20, F21, F22, and F23, it is evident that until the final iterations, the algorithm convergence process as well as population diversity continues. In handling the F5 function, it is evident that the convergence process continues until the final iteration. In the optimization of function F6, SSVUBA with high search power reached the global optimization, and then the population diversity decreased. In the optimization of function F10, the population diversity decreased while the algorithm achieved an acceptable solution. In solving function F11, the population diversity decreased, while SSVUBA converged to the best solution, the global optima. In optimizing function F14, the population diversity decreased after the algorithm converged to the optimal solution. Therefore, the results of population diversity analysis indicate the high ability of SSVUBA in maintaining population diversity, which has led to its effective performance in providing appropriate solutions for objective functions.

### 4.7. Evaluation of the CEC 2017 Test Functions

In this subsection, the performance of SSVUBA in addressing the CEC 2017 benchmark is examined. The CEC 2017 set includes three unimodal functions (C1 to C3), seven simple multimodal functions (C4 to C10), ten hybrid functions (C11 to C20), and ten composition functions (C21 to C30). The results obtained from the implementation of SSVUBA and competitor algorithms for these functions are shown in Table 9. What can be deduced from the simulation results is that SSVUBA performed better than competitor algorithms in handling the C1, C2, C4, C5, C11, C12, C13, C14, C15, C16, C17, C18, C19, C20, C21, C24, C26, C27, C29, and C30 functions.

## 5. Discussion

Two essential factors that influence the performance of optimization algorithms are the exploitation and exploration capabilities. To give an acceptable solution to an optimization issue, each optimization algorithm must strike a reasonable balance between these two requirements.

In the study of optimization algorithms, the idea of exploitation refers to the algorithm’s capacity to search locally. In reality, after reaching the optimal area in the optimization problem’s search space, an optimization algorithm should be able to converge as much as feasible to the global optimal. As a result, when comparing the performance of several algorithms in solving an optimization issue, an algorithm that provides a solution that is closer to the global optimal has a better exploitation capability. The exploitation ability of an algorithm is essential, especially when solving problems that have only one basic solution. The objective functions F1 to F7, which are unimodal functions, have the property that they lack local optimal solutions and have only one main solution. As a result, functions F1 to F7 are good candidates for testing the exploitation ability of optimization techniques. The optimization results of the unimodal objective functions reported in Table 2 show that the proposed SSVUBA has a higher capability in local search than the compared algorithms and with high exploitation power, is able to deliver solutions very close to the global optimal.

In the study of optimization algorithms, the idea of exploration refers to the algorithm’s capacity to search globally. In reality, to find the optimal area, an optimization algorithm should be able to correctly scan diverse portions of the search space. Exploration power enables the algorithm to pass through all optimal local areas and avoid becoming trapped in a local optimum. As a result, when comparing the potential of various optimization algorithms to handle an optimization issue, an algorithm that can appropriately check the problem search space to distance itself from all local optimal solutions and move towards the global optimal solution has a higher exploration ability. The exploration ability of an algorithm is of particular importance, especially when solving issues with several optimal local solutions in addition to the original solution. The objective functions F8 to F23, which are multimodal functions, have this feature. As a result, these functions are good candidates for testing the exploration ability in optimization algorithms. The examination of the results of optimization of multimodal functions, provided in Table 3 and Table 4, shows that the SSVUBA has a superior ability in global search and is capable of passing through the local optimum areas due to its high exploration power.

Although exploitation and exploration affect the performance of optimization algorithms, each alone is not enough for the algorithm to succeed in optimization. Therefore, there is a need for a balance between these two indicators for an algorithm to be able to handle optimization problems. The simulation results show that SSVUBA has a high potential for balancing exploration and exploitation. The superiority of SSVUBA in the management of optimization applications with statistical criteria and ranking compared to competitor algorithms is evident. However, statistical analysis of the Wilcoxon rank sum test shows that this superiority is also statistically significant.

SSVUBA sensitivity analysis to parameters N and T shows that the performance of the proposed algorithm under the influence of changes in these two parameters provides different results. This is because the algorithm must have sufficient power to scan the search space whose tool is search agents (population members, i.e., N), as well as a sufficient opportunity (i.e., T) to identify the optimal area and converge towards the global optima. Thus, as expected, increasing the T and N values improved the SSVUBA performance and decreased the target function values.

To further analyze the performance of SSVUBA in optimization applications, this proposed method, along with competitor algorithms, was implemented on the CEC 2017 test suite. The simulation results in this type of optimization challenge indicate the successful performance of SSVUBA in addressing this type of optimization problem. Comparing SSVUBA with competing algorithms, it was found that SSVUBA ranked first in most cases and was more efficient than the compared algorithms.

## 6. SSVUBA for Engineering Design Applications

In order to analyze the efficiency of SSVUBA in real world purposes, this optimizer has been employed to address four engineering problems: pressure vessel design, speed reducer design, welded beam design, and tension/compression spring design.

### 6.1. Pressure Vessel Design Problem

**Figure 8:**
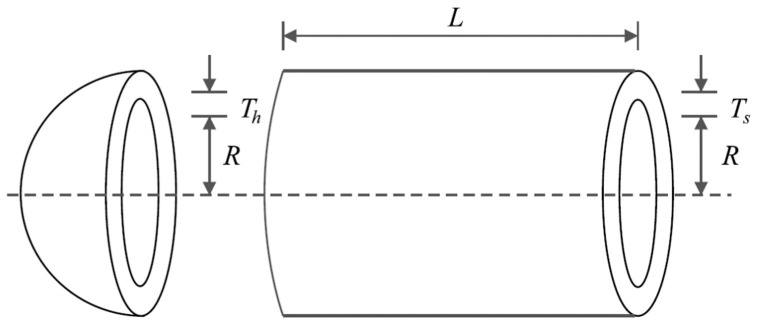
Schematic of the pressure vessel design.

Pressure vessel design is an engineering challenge in which the design purpose is minimizing the total cost (material, forming, and welding) of the cylindrical pressure vessel [41]. The schematic of this issue is shown in Figure 8. This problem’s mathematical model is as follows:

Consider: $X=\left[x_{1},\;x_{2},\;x_{3},\;x_{4}\right]=\left[T_{s},\;T_{h},\;R,\;L\right]$.

Minimize: $f\left(x\right)=0.6224x_{1}x_{3}x_{4}+1.778x_{2}x_{3}^{2}+3.1661x_{1}^{2}x_{4}+19.84x_{1}^{2}x_{3}.$

Subject to:

$g_{1}\left(x\right)=-x_{1}+0.0193x_{3}\;\leq \;0,$

$g_{2}\left(x\right)=-x_{2}+0.00954x_{3}\leq \;0,$

$g_{3}\left(x\right)=-\pi x_{3}^{2}x_{4}-\frac{4}{3}\pi x_{3}^{3}+1,296,000\leq \;0,$

$g_{4}\left(x\right)=x_{4}-240\;\leq \;0.$

With

$$0\leq x_{1},x_{2}\leq 100,\;\;\mathrm{and}\;10\leq x_{3},x_{4}\leq 200.$$

**Figure 9:**
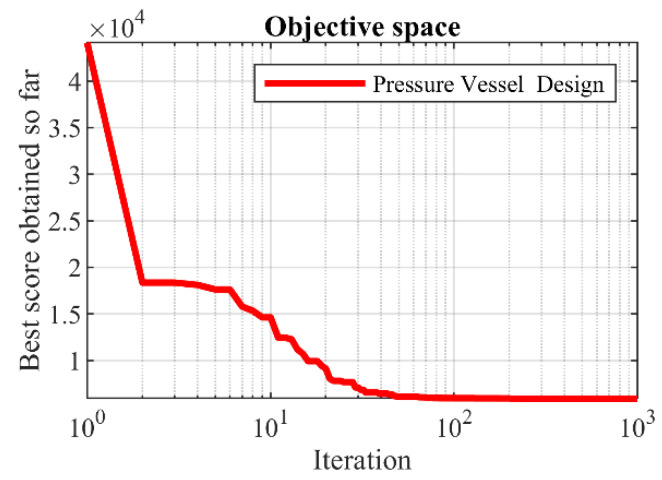
SSVUBA’s performance convergence curve in the pressure vessel design.

The implementation results of SSVUBA and eight competitor algorithms in achieving the optimal design for pressure vessel are reported in Table 10. SSVUBA presents the optimal solution with the values of the variables equal to (0.7789938, 0.3850896, 40.3607, 199.3274) and the value of the objective function (5884.8824). The statistical results of SSVUBA performance against eight competitor algorithms in optimizing the pressure vessel problem are presented in Table 11. What can be seen from the statistical results is that SSVUBA has a superior performance over the compared algorithms by providing better values in statistical indicators. The behavior of the SSVUBA convergence curve during achieving the optimal solution for pressure vessel design is presented in Figure 9.

### 6.2. Speed Reducer Design Problem

**Figure 10:**
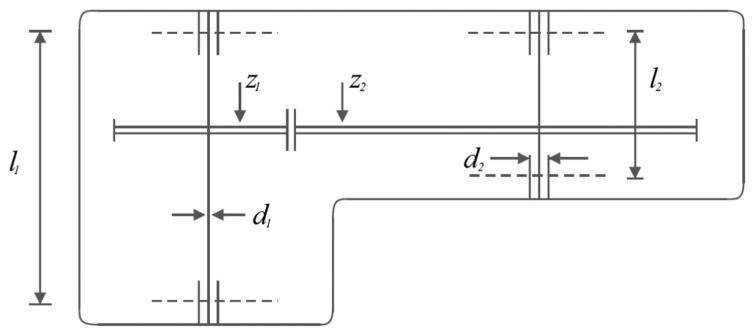
Schematic of the speed reducer design.

Speed reducer design is a minimization challenge whose main goal in optimal design is to reduce the weight of the speed reducer, which is depicted schematically in Figure 10 [42,43]. This problem’s mathematical model is as follows:

Consider: $X=\left[x_{1,}\;x_{2},\;x_{3},\;x_{4},\;x_{5}\;,x_{6}\;,x_{7}\right]=\left[b,\;m,\;p,\;l_{1},\;l_{2},\;d_{1},\;d_{2}\right]$.

Minimize: $f\left(x\right)=0.7854x_{1}x_{2}^{2}\left(3.3333x_{3}^{2}+14.9334x_{3}-43.0934\right)-1.508x_{1}\left(x_{6}^{2}+x_{7}^{2}\right)+7.4777\left(x_{6}^{3}+x_{7}^{3}\right)+0.7854\left(x_{4}x_{6}^{2}+x_{5}x_{7}^{2}\right)$.

Subject to:

$g_{1}\left(x\right)=\frac{27}{x_{1}x_{2}^{2}x_{3}}-1\;\leq \;0,$

$g_{2}\left(x\right)=\frac{397.5}{x_{1}x_{2}^{2}x_{3}}-1\leq \;0,$

$g_{3}\left(x\right)=\frac{1.93x_{4}^{3}}{x_{2}x_{3}x_{6}^{4}}-1\leq \;0,$

$g_{4}\left(x\right)=\frac{1.93x_{5}^{3}}{x_{2}x_{3}x_{7}^{4}}-1\;\leq \;0,$

$g_{5}\left(x\right)=\frac{1}{110x_{6}^{3}}\sqrt{{\left(\frac{745x_{4}}{x_{2}x_{3}}\right)}^{2}+16.9\times 10^{6}}-1\leq \;0,$

$g_{6}\left(x\right)=\frac{1}{85x_{7}^{3}}\sqrt{{\left(\frac{745x_{5}}{x_{2}x_{3}}\right)}^{2}+157.5\times 10^{6}}-1\;\leq \;0,$

$g_{7}\left(x\right)=\frac{x_{2}x_{3}}{40}-1\;\leq \;0,$

$g_{8}\left(x\right)=\frac{5x_{2}}{x_{1}}-1\;\leq \;0,$

$g_{9}\left(x\right)=\frac{x_{1}}{12x_{2}}-1\;\leq \;0,$

$g_{10}\left(x\right)=\frac{1.5x_{6}+1.9}{x_{4}}-1\;\leq \;0,$

$g_{11}\left(x\right)=\frac{1.1x_{7}+1.9}{x_{5}}-1\;\leq \;0.$

With

$$2.6\leq x_{1}\leq 3.6,\;0.7\leq x_{2}\leq 0.8,\;17\leq x_{3}\leq 28,\;7.3\leq x_{4}\leq 8.3,\;7.8\leq x_{5}\;\;\;\;\leq 8.3,\;2.9\leq x_{6}\leq 3.9,\;\;\;\mathrm{and}\;\;5\leq x_{7}\leq 5.5\;.$$

**Figure 11:**
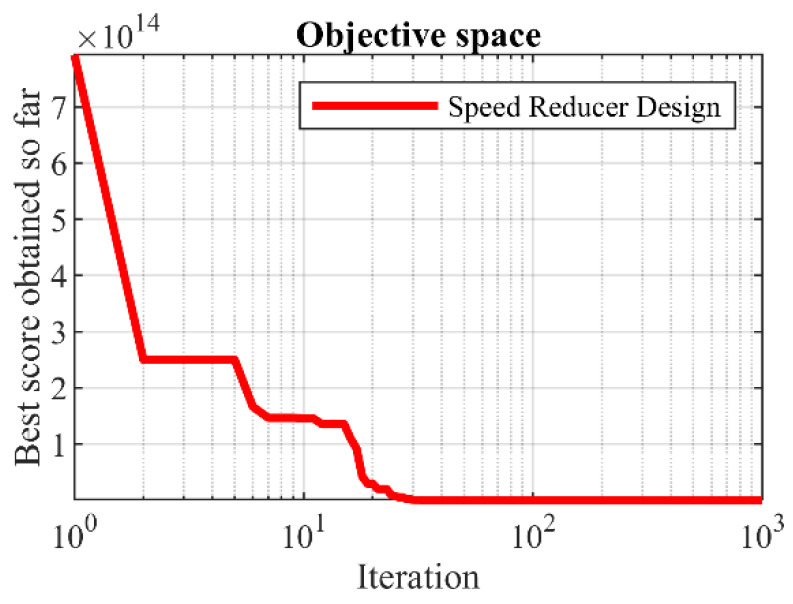
SSVUBA’s performance convergence curve in the speed reducer design.

The results obtained from SSVUBA and eight competing algorithms in optimizing the speed reducer design are presented in Table 12. Based on the simulation results, it is obvious that SSVUBA has provided the optimal design of this problem for the values of the variables equal to (3.50003, 0.700007, 17, 7.3, 7.8, 3.35021, 5.28668) and the value of the objective function equal to (2996.3904). The statistical results of the SSVUBA performance as well as competitor algorithms in optimizing the speed reducer problem are reported in Table 13. Statistical results show the superiority of SSVUBA over competitor algorithms. The SSVUBA convergence curve when solving the speed reducer design is shown in Figure 11.

### 6.3. Welded Beam Design

**Figure 12:**
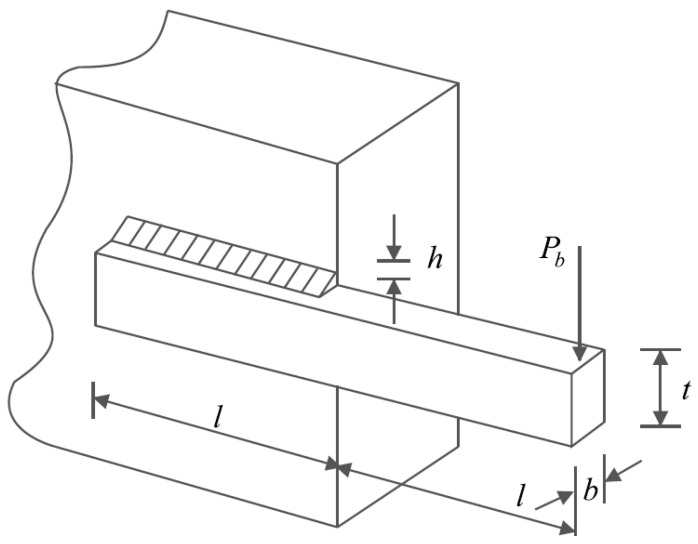
Schematic of the welded beam design.

Welded beam design is an engineering topic with the main goal of minimizing the fabrication cost of the welded beam, a schematic of which is shown in Figure 12 [26]. This problem’s mathematical model is as follows:

Consider: $X=\left[x_{1},\;x_{2},\;x_{3},\;x_{4}\right]=\left[h,\;l,\;t,\;b\right]$.

Minimize: $f\;\left(x\right)=1.10471x_{1}^{2}x_{2}+0.04811x_{3}x_{4}\;\left(14.0+x_{2}\right)$.

Subject to:

$g_{1}\;\left(x\right)=\tau \left(x\right)-13,600\;\leq \;0,$

$g_{2}\;\left(x\right)=\sigma \left(x\right)-30,000\;\leq \;0,$

$g_{3}\;\left(x\right)=x_{1}-x_{4}\leq \;0,$

$g_{4}\;\left(x\right)=0.10471x_{1}^{2}+0.04811x_{3}x_{4}\;\left(14+x_{2}\right)-5.0\;\leq \;0,$

$g_{5}\left(x\right)=0.125-x_{1}\leq \;0,$

$g_{6}\left(x\right)=\delta \;\left(x\right)-0.25\;\leq \;0,$

$g_{7}\;\left(x\right)=6000-p_{c}\;\left(x\right)\;\leq \;0.$

where

$\tau \left(x\right)=\sqrt{\tau^{\prime }+\left(2\tau \tau^{\prime }\right)\frac{x_{2}}{2R}+{\left(\tau ”\right)}^{2}},$

$\tau^{\prime }=\frac{6000}{\sqrt{2}x_{1}x_{2}},$

$\tau ”=\frac{MR}{J},$

$M=6000\left(14+\frac{x_{2}}{2}\right),$

$R=\sqrt{\frac{x_{2}^{2}}{4}+{\left(\frac{x_{1}+x_{3}}{2}\right)}^{2}},$

$J=2\left\{x_{1}x_{2}\sqrt{2}\left[\frac{x_{2}^{2}}{12}+{\left(\frac{x_{1}+x_{3}}{2}\right)}^{2}\right]\right\},$

$\sigma \left(x\right)=\frac{50,4000}{x_{4}x_{3}^{2}}$

$\delta \;\left(x\right)=\frac{65,856,000}{\left(30\cdot 10^{6}\right)x_{4}x_{3}^{3}},$

$p_{c}\;\left(x\right)=\frac{4.013\left(30\cdot 10^{6}\right)\sqrt{\frac{x_{3}^{2}x_{4}^{6}}{36}}}{196}\left(1-\frac{x_{3}}{28}\sqrt{\frac{30\cdot 10^{6}}{4\left(12\cdot 10^{6}\right)}}\right).$

With

$$0.1\leq x_{1},\;x_{4}\leq 2\;\mathrm{and}\;0.1\leq x_{2},\;x_{3}\leq 10.$$

**Figure 13:**
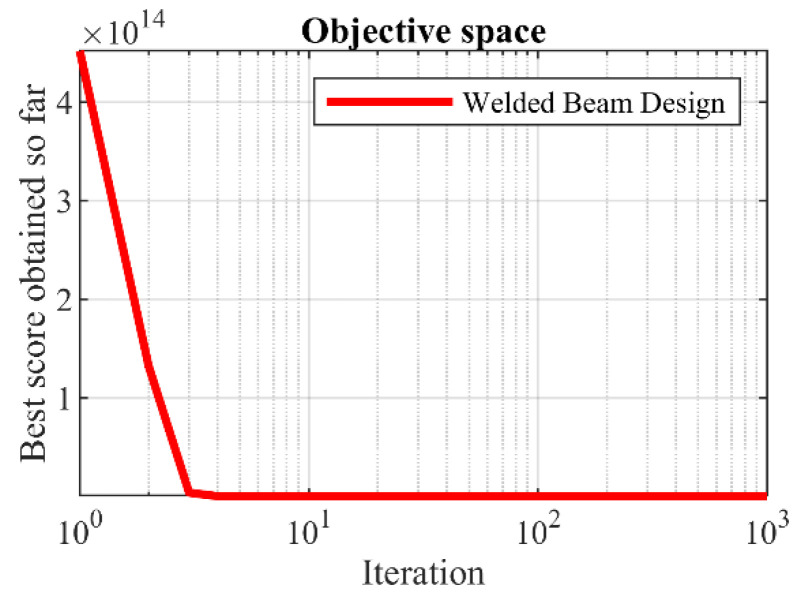
SSVUBA’s performance convergence curve for the welded beam design.

The optimization results for the welded beam design are reported in Table 14. Analysis of the simulation results shows that SSVUBA has provided the optimal design for the welded beam with the values of the variables equal to (0.205730, 3.4705162, 9.0366314, 0.2057314) and the value of the objective function equal to (1.724852). The statistical results obtained from the implementation of SSVUBA and eight competitor algorithms on this design are presented in Table 15. Analysis of the results of this table shows that SSVUBA with better values in statistical indicators provides superior performance in solving the welded beam design against competitor algorithms. The SSVUBA convergence curve for the optimal solution of the welded beam design problem is shown in Figure 13.

### 6.4. Tension/Compression Spring Design Problem

**Figure 14:**
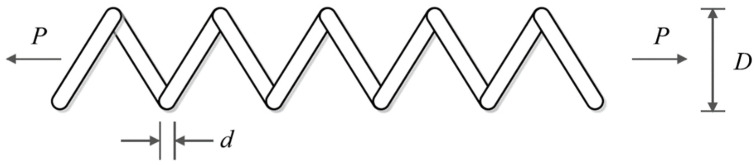
Schematic of the tension/compression spring design.

Tension/compression spring design is an engineering challenge aimed at reducing the weight of the tension/compression spring, a schematic of which is shown in Figure 14 [26]. This problem’s mathematical model is as follows:

Consider$:\;X=\left[x_{1},\;x_{2},\;x_{3}\;\right]=\left[d,\;D,\;P\right].$

Minimize: $f\;\left(x\right)=\left(x_{3}+2\right)x_{2}x_{1}^{2}.$

Subject to:

$g_{1}\;\left(x\right)=1-\frac{x_{2}^{3}x_{3}}{71,785x_{1}^{4}}\;\leq \;0,$

$g_{2}\;\left(x\right)=\frac{4x_{2}^{2}-x_{1}x_{2}}{12,566\left(x_{2}x_{1}^{3}\right)}+\frac{1}{5108x_{1}^{2}}-1\leq \;0,$

$g_{3}\;\left(x\right)=1-\frac{140.45x_{1}}{x_{2}^{2}x_{3}}\leq \;0$ *,*

$g_{4}\;\left(x\right)=\frac{x_{1}+x_{2}}{1.5}-1\;\leq \;0$.

With

$$0.05\leq x_{1}\leq 2,\;0.25\leq x_{2}\leq 1.3\;\;\mathrm{and}\;\;2\leq \;x_{3}\leq 15.$$

**Figure 15:**
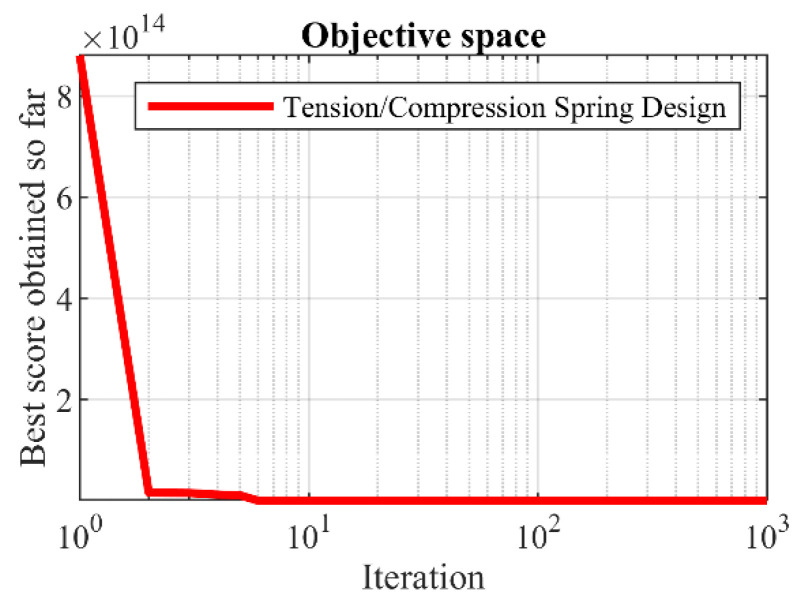
SSVUBA’s performance convergence curve for the tension/compression spring.

The results for the tension/compression spring design variables using SSVUBA and compared methods are provided in Table 16. The simulation results reveal that SSVUBA provides the optimal solution with the values of the variables equal to (0.051704, 0.357077, 11.26939) and the value of the objective function equal to (0.012665). The statistical results of implementation of SSVUBA and compared algorithms for the tension/compression spring problem are presented in Table 17. The observations indicate the superiority of SSVUBA performance due to the provision of better values of statistical indicators compared to competitor algorithms. The SSVUBA convergence curve when achieving the optimal solution to the tension/compression spring problem is shown in Figure 15.

### 6.5. The SSVUBA’s Applicability in Sensor Networks and Image Processing

Many complex problems in the field of image processing are the focus of extensive research to find efficient methods. In this subject, local search approaches are commonly utilized for solving difficult problems. However, many issues and research in image processing are combinatorial and NP-hard. As optimization algorithms are population-based stochastic approaches, they are generally better suited to solving these complicated challenges. As a result, optimization algorithms such as proposed SSVUBA can prevent becoming stuck in the local optimum and can frequently locate the global optimal solution. Recent advancements have resulted in an increased use of artificial intelligence approaches for image processing. Today, wireless sensor networks are one of the most popular wireless networks due to their various applications. These networks consist of a set of automated sensors to monitor physical or environmental conditions such as heat, sound, vibration, pressure, motion, or pollution. As a result, sensor networks are faced with a huge amount of valuable information. In this type of application, data analysis using classical methods is not very efficient and appropriate. Because of this, artificial intelligence approaches, such as the employment of the proposed SSVUBA for various applications in image processing and sensor networks, have become significant. The proposed SSVUBA approach is effective for topics such as energy optimization in sensor networks, sensor network placement, network coding (NC) in wireless sensor networks, sensor network coverage optimization, clustering in sensor networks, medical image processing, pattern recognition, video processing, and so on.

## 7. Conclusions and Future Works

Numerous optimization issues have been defined in various disciplines of science that must be addressed by employing proper approaches. One of the most successful and extensively used approaches for tackling such issues is optimization algorithms, which belong to the category of random methods. To handle different optimization challenges, a novel optimization technique named “Selecting Some Variables to Update-Based Algorithm” (SSVUBA) was developed in this study. Making more use of the information of different members of the population and adjusting the number of selected variables in order to update the population of the algorithm during successive iterations of the algorithm were the main ideas in the design of the proposed SSVUBA. The ability of SSVUBA to solve optimization problems was tested on fifty-three different objective functions. The results of optimization of unimodal functions indicated the strong ability of the proposed algorithm in the exploitation index and the presentation of solutions very close to the global optimal. The optimization results of multi-model functions showed that the SSVUBA with high capability in the exploration index is able to scan the search space of the problem and accurately and converge to the global optimal by passing local optimal areas. Further, in order to analyze the optimization results obtained from SSVUBA, these results were compared with the performance of eight well-known algorithms: PSO, TLBO, GWO, WOA, MPA, TSA, GSA, GA, RFO, RSA, AHA, and HBA. What is clear from the analysis of simulation results is that the SSVUBA has a strong ability to solve optimization problems by providing appropriate quasi-optimal solutions, and its performance is superior and more competitive than that of similar algorithms. In order to further analyze SSVUBA in optimization, the proposed algorithm was employed to optimize four engineering design challenges. The optimization results indicated the effective performance of SSVUBA in real-world applications and the provision of optimal values for design variables.

The authors provide various recommendations for future research, including the development of multi-objective and binary SSVUBA versions. Other proposals for future investigations of this work include using the proposed SSVUBA to solve optimization issues in many fields as well as real-world applications. The proposed SSVUBA approach opens up a wide range of future studies. These studies include the SSVUBA employment in wireless sensor networks, image processing, machine learning, signal denoising, artificial intelligence, engineering, feature selection, big data, data mining, and other optimization chalenges.

As with all stochastic approaches for optimization problems, the limitation of the proposed SSVUBA is that it offers no guarantee that the solutions provided by it will be the global optimal. Another limitation of any random approach, including SSVUBA, is that it is always possible for researchers to develop new algorithms that can provide more effective solutions to optimization issues. Moreover, according to the NFL theorem, another limitation of SSVUBA is that its strong performance in solving a group of optimization applications leaves no reason to offer the same performance in all other optimization applications.

## Figures and Tables

**Table 1:** Parameter values for the compared algorithms.

Algorithm	Parameter	Value
HBA	The ability of a honey badger to get food	β=6
	Constant number	*C* = 2
AHA		
	Migration coefficient	2*N* (*N* is the population size)
RSA		
	Sensitive parameter	β=0.01
	Sensitive parameter	α=0.1
	Evolutionary Sense (ES)	ES: randomly decreasing values between 2 and −2
RFO		
	$\mathrm{Fox}\;\mathrm{observation}\;\mathrm{angle}\;(\varphi_{0}$)	$\varphi_{0}\in \left(0,\;2\pi \right)$
	Weather conditions (θ)	random value between 0 and 1
	Scaling parameter	a∈(0, 0.2)
MPA		
	Constant number	*P* = 0.5
	Random vector	*R* ∈ [0, 1]
	Fish-Aggregating Devices (*FADs*)	*FADs* = 0.2
	Binary vector	*U* = 0 or 1
TSA		
	P_min_	1
	P_max_	4
	$c_{1},c_{2},c_{3}$	random numbers in the interval [0, 1].
WOA		
	*a*: Convergence parameter	Linear reduction from 2 to 0.
	*r*: random vector	r ∈ [0, 1].
	*l*: random number	*l* ∈ [−1, 1].
GWO		
	Convergence parameter (*a*)	*a*: Linear reduction from 2 to 0.
TLBO		
	*T_F_*: teaching factor	$T_{F}=\mathrm{round}\;\left[\left(1+rand\right)\right]$
	random number	*rand* ∈ [0, 1].
GSA		
	Alpha	20
	*R_power_*	1
	*R_norm_*	2
	*G_0_*	100
PSO		
	Topology	Fully connected
	Cognitive constant	$C_{1}=2$
	Social constant	$C_{2}=2$
	Inertia weight	Linear reduction from 0.9 to 0.1
	Velocity limit	10% of variables’ dimension range
GA		
	Type	Real coded
	Selection	Roulette wheel (Proportionate)
	Crossover	Whole arithmetic (Probability = 0.8, α∈[−0.5, 1.5])
	Mutation	Gaussian (Probability = 0.05)

**Table 2:** Assessment results of unimodal functions.

		GA	PSO	**GSA**	TLBO	GWO	WOA	TSA	MPA	RFO	RSA	AHA	HBA	SSVUBA
F_1_	avg	13.22731	1.77 × 10^−5^	2.02 × 10^−17^	1.33 × 10^−59^	1.09 × 10^−58^	1.79 × 10^−64^	8.2 × 10^−33^	1.7 × 10^−18^	6.46 × 10^−84^	3.1 × 10^−126^	2.8 × 10^−140^	4.77 × 10^−75^	5.02 × 10^−185^
	std	5.72164	5.85 × 10^−5^	7.09 × 10^−18^	2.05 × 10^−59^	4.09 × 10^−58^	2.75 × 10^−64^	2.53 × 10^−32^	6.75 × 10^−18^	2.64 × 10^−83^	1.3 × 10^−125^	1.1 × 10^−139^	1.41 × 10^−74^	1.72 × 10^−665^
	bsf	5.587895	2 × 10^−10^	8.19 × 10^−18^	9.35 × 10^−61^	7.72 × 10^−61^	1.25 × 10^−65^	1.14 ×10^−62^	3.41 × 10^−28^	9.43 × 10^−93^	1 × 10^−132^	3.6 × 10^−166^	5.24 × 10^−81^	9.98 × 10^−193^
	med	11.03442	9.91 × 10^−7^	1.78 × 10^−17^	4.69 × 10^−60^	1.08 × 10^−59^	6.28 × 10^−65^	3.89 × 10^−38^	1.27 × 10^−19^	3.69 × 10^−88^	5.3 × 10^−129^	7.4 × 10^−150^	2.45 × 10^−76^	2.22 × 10^−189^
	rank	13	12	11	7	8	6	9	10	4	3	2	5	1
F_2_	avg	2.476931	0.340796	2.37 × 10^−8^	5.54 × 10^−35^	1.29 × 10^−34^	1.57 × 10^−51^	5.01 × 10^−39^	2.78 × 10^−9^	6.78 × 10^−46^	1.31 × 10^−66^	1.07 × 10^−74^	3.84 × 10^−40^	1.60 × 10^−99^
	std	0.642211	0.668924	3.96 × 10^−9^	4.7 × 10^−35^	2.2 × 10^−34^	5.94 × 10^−51^	1.72 × 10^−38^	1.08 × 10^−8^	1.51 × 10^−45^	5.02 × 10^−66^	2.83 × 10^−74^	1.25 × 10^−39^	2.68 × 10^−99^
	bsf	1.589545	0.00174	1.59 × 10^−8^	1.32 × 10^−35^	1.54 × 10^−35^	1.14 × 10^−57^	8.25 × 10^−43^	4.25 × 10^−18^	4.79 × 10^−49^	4.81 × 10^−71^	1.59 × 10^−85^	2.28 × 10^−43^	3.41 × 10^−101^
	med	2.46141	0.129983	2.33 × 10^−8^	4.37 × 10^−35^	6.37 × 10^−35^	1.89 × 10^−54^	8.25 × 10^−41^	3.18 × 10^−11^	3.56 × 10^−47^	1.33 × 10^−68^	2.45 × 10^−78^	1.73 × 10^−41^	6.87 × 10^−100^
	rank	13	12	11	8	9	4	7	10	5	3	2	6	1
F_3_	avg	1535.359	588.9025	279.0646	7 × 10^−15^	7.4 × 10^−15^	7.55 × 10^−9^	3.19 × 10^−19^	0.37663	4.76 × 10^−58^	4.62 × 10^−84^	5.9 × 10^−128^	9.05 × 10^−51^	2.01 × 10^−154^
	std	366.8302	1522.483	112.1922	1.27 × 10^−14^	1.9 × 10^−14^	2.38 × 10^−9^	9.89 × 10^−19^	0.20155	1.3 × 10^−57^	2.07 × 10^−83^	2 × 10^−127^	3.54 × 10^−50^	8.97 × 10^−154^
	bsf	1013.675	1.613322	81.8305	1.21 × 10^−16^	4.74 × 10^−20^	3.38 × 10^−9^	7.28 × 10^−30^	0.032006	1.19 × 10^−69^	5.8 × 10^−100^	8.3 × 10^−162^	1.2 × 10^−57^	3.29 × 10^−169^
	med	1509.204	54.1003	291.1394	1.86 × 10^−15^	1.59 × 10^−16^	7.19 × 10^−9^	9.8 × 10^−21^	0.378279	1.49 × 10^−61^	2.61 × 10^−94^	2.1 × 10^−138^	1.39 × 10^−54^	7.70 × 10^−162^
	rank	13	12	11	7	8	9	6	10	4	3	2	5	1
F_4_	avg	2.092152	3.959462	3.25× 10^−9^	1.58 × 10^−15^	1.26 × 10^−14^	0.001283	2.01 × 10^−22^	3.66×10^−8^	1.34 × 10^−35^	9.09 × 10^−52^	5.93 × 10^−57^	2.65 × 10^−31^	6.62 × 10^−59^
	std	0.336658	2.201879	7.49× 10^−10^	7.13 × 10^−16^	2.32 × 10^−14^	0.00062	5.96 × 10^−22^	6.44 × 10^−8^	3.82 × 10^−35^	3.17 × 10^−51^	2.65 × 10^−56^	5.17 × 10^−31^	1.76 × 10^−58^
	bsf	1.388459	1.602806	2.09× 10^−9^	6.41 × 10^−16^	3.43 × 10^−16^	5.87 × 10^−5^	1.87 × 10^−52^	3.42 × 10^−17^	3.83 × 10^−40^	5.65 × 10^−57^	2.83 × 10^−60^	2.98 × 10^−34^	1.43 × 10^−63^
	med	2.096441	3.257411	3.34× 10^−9^	1.54 × 10^−15^	7.3 × 10^−15^	0.001416	3.13 × 10^−27^	3.03 × 10^−8^	2.7 × 10^−37^	5.77 × 10^−55^	1 × 10^−58^	3.55 × 10^−32^	4.27 × 10^−60^
	rank	12	13	9	7	8	11	6	10	4	3	1	5	1
F_5_	avg	310.1169	50.2122	36.07085	145.5196	26.83384	27.14826	28.73839	42.45484	27.45887	28.69673	26.65474	26.68016	2.54 × 10^−12^
	std	120.3226	36.48688	32.43014	19.72018	0.883186	0.627034	0.364483	0.614622	0.72896	0.651915	0.41764	1.008602	1.08 × 10^−21^
	bsf	160.3408	3.643404	25.81227	120.6724	25.1868	26.40605	28.50977	41.54523	26.21217	27.0064	26.08727	25.11442	3.16 × 10^−24^
	med	279.2378	28.66429	26.04868	142.7508	26.68203	26.9085	28.5106	42.44818	27.18532	28.98402	26.64571	26.51364	2.60 × 10^−17^
	rank	13	11	9	12	4	5	8	10	6	7	2	3	1
F_6_	avg	14.53545	20.22975	0	0.44955	0.641682	0.071455	3.84 × 10^−20^	0.390478	1.54416	6.901619	0	0.646884	0
	std	5.829403	12.76004	0	0.509907	0.300774	0.078108	1.5 × 10^−19^	0.080203	0.399298	0.87614	0	0.27258	0
	bsf	5.994	4.995	0	0	1.57 × 10^−5^	0.014631	6.74 × 10^−26^	0.274307	0.862897	3.58704	0	0.015007	0
	ed	13.4865	18.981	0	0	0.620865	0.029288	6.74 × 10^−21^	0.406241	1.639428	7.210589	0	0.674911	0
	rank	10	11	1	5	6	3	2	4	8	9	1	7	1
F_7_	avg	0.005674	0.1133	0.020671	0.003127	0.000819	0.001928	0.000276	0.00218	0.000401	0.000147	0.000304	0.00019	9.00 × 10^−5^
	std	0.00243	0.04582	0.011349	0.00135	0.000503	0.003338	0.000123	0.000466	0.000307	0.000169	0.000268	0.000257	6.34 × 10^−25^
	bsf	0.002109	0.029564	0.01005	0.00136	0.000248	4.24 × 10^−5^	0.000104	0.001428	2.99 × 10^−05^	1.24 × 10^−05^	2.81 × 10^−06^	3.96 × 10^−06^	7.75 × 10^−6^
	med	0.005359	0.107765	0.016978	0.002909	0.000629	0.000979	0.000367	0.002178	0.000317	8.1 × 10^−05^	0.000182	0.000104	7.75 × 10^−5^
	rank	11	13	12	10	7	8	4	9	6	2	5	3	1
Sum rank		85	84	64	56	50	46	42	63	37	30	15	34	7
Mean rank		12.1428	12	9.1428	8	7.1428	6.5714	6	9	5.2857	4.2857	2.1428	4.8571	1
Total rank		13	12	11	9	8	7	6	10	5	3	2	4	1

**Table 3:** Assessment results of high-dimensional multimodal functions.

		GA	PSO	GSA	TLBO	GWO	WOA	TSA	MPA	RFO	AHA	RSA	HBA	SSVUBA
F_8_	avg	−8176.2	−6901.75	−2846.22	−7795.8	−5879.23	−7679.85	−5663.98	−3648.49	−7548.39	−5281.28	−11,102.4	−8081.04	−12,569.5
	std	794.342	835.8931	539.8674	985.735	983.5375	1103.956	21.87234	474.1073	1154.307	563.2137	578.0354	968.1117	1.87 × 10^−22^
	bsf	−9708.0	−8492.94	−3965.26	−9094.7	−7219.83	−8588.51	−5700.59	−4415.48	−9259.4	−5647.03	−12,173.2	−10,584.1	−12,569.5
	med	−8109.5	−7091.86	−2668.65	−7727.5	−5768.85	−8282.39	−5663.96	−3629.21	−7805.26	−5508.56	−11,135.5	−8049.62	−12,569.5
	rank	3	8	13	5	9	6	10	12	7	11	2	4	1
F_9_	avg	62.349	57.0043	16.25131	10.6668	8.52 × 10^−15^	0	0.005882	152.539	0	0	0	0	0
	std	15.2006	16.50103	4.654009	0.39675	2.08 × 10^−14^	0	0.000696	15.16653	0	0	0	0	0
	bsf	36.8294	27.83098	4.96982	9.86409	0	0	0.004772	128.1024	0	0	0	0	0
	med	61.6169	55.16946	15.40644	10.8757	0	0	0.005865	154.4667	0	0	0	0	0
	rank	7	6	5	4	2	1	3	8	1	1	1	1	1
F_10_	avg	3.21861	2.152524	3.56 × 10^−9^	0.26294	1.7 × 10^−14^	3.9 × 10^−15^	6.4 × 10^−11^	8.3 × 10^−10^	4.5 × 10^−13^	8.9 × 10^−16^	8.9 × 10^−16^	7.1 × 10^−13^	8.9 × 10^−16^
	std	0.36141	0.548903	5.3 × 10^−10^	0.07279	3.2 × 10^−15^	2.6 × 10^−15^	2.6 × 10^−10^	2.8 × 10^−9^	2.0 × 10^−12^	0	0	3.2 × 10^−12^	0
	bsf	2.75445	1.153996	2.6 × 10^−9^	0.15615	1.5 × 10^−14^	8.9 × 10^−16^	8.1 × 10^−15^	1.7 × 10^−18^	8.9 × 10^−16^	8.9 × 10^−16^	8.9 × 10^−16^	8.9 × 10^−16^	8.9 × 10^−16^
	med	3.1172	2.167913	3.63 × 10^−9^	0.26128	1.5 × 10^−14^	4.4 × 10^−15^	1.09 × 10^−13^	1.1 × 10^−11^	8.9 × 10^−16^	8.9 × 10^−16^	8.9 × 10^−16^	8.9 × 10^−16^	8.9 × 10^−16^
	rank	11	10	8	9	3	2	6	7	4	1	1	5	1
F_11_	avg	1.228978	0.046246	3.733827	0.587096	0.003749	0.003017	1.54 × 10^−6^	0	0	0	0	0	0
	std	0.062697	0.051782	1.66862	0.16895	0.007337	0.013494	3.38 × 10^−6^	0	0	0	0	0	0
	bsf	1.139331	7.28 × 10^−9^	1.517769	0.309807	0	0	4.23 × 10^−15^	0	0	0	0	0	0
	med	1.226004	0.029444	3.420843	0.581444	0	0	8.76 × 10^−7^	0	0	0	0	0	0
	rank	7	5	8	6	4	3	2	1	1	1	1	1	1
F_12_	avg	0.046979	0.480186	0.036247	0.020531	0.037173	0.007721	0.050113	0.082476	0.069238	1.275979	0.000916	0.016112	1.62 × 10^−32^
	std	0.028455	0.601971	0.060805	0.028617	0.013862	0.008975	0.009845	0.002384	0.039794	0.318983	0.001997	0.007672	2.16 × 10^−33^
	bsf	0.018345	0.000145	5.57 × 10^−20^	0.002029	0.019275	0.001141	0.035393	0.077834	0.012096	0.595234	5.91 × 10^−5^	0.000811	1.57 × 10^−32^
	med	0.041748	0.155444	1.48 × 10^−19^	0.015166	0.032958	0.003915	0.050884	0.082026	0.061529	1.368211	0.000229	0.017314	1.57 × 10^−32^
	rank	8	12	6	5	7	3	9	11	10	13	2	4	1
F_13_	avg	1.207336	0.507903	0.002083	0.328792	0.575742	0.1931	2.656091	0.564683	1.803955	0.454655	2.113078	1.253473	7.65 × 10^−32^
	std	0.333421	1.25043	0.00547	0.198741	0.170178	0.150736	0.009777	0.187631	0.41072	0.922164	0.416593	0.460513	1.61 × 10^−31^
	bsf	0.497592	9.98 × 10^−7^	1.18 × 10^−18^	0.038228	0.297524	0.029632	2.629118	0.280015	1.051985	1.22 × 10^−19^	1.063506	0.547271	1.35 × 10^−32^
	med	1.216834	0.043953	2.14 × 10^−18^	0.282482	0.577744	0.151854	2.659088	0.579275	1.694537	8.11 × 10^−14^	2.100496	1.258265	1.35 × 10^−32^
	rank	9	6	2	4	8	3	13	7	11	5	12	10	1
Sum rank		45	47	42	33	33	18	43	46	34	32	19	25	6
Mean rank		7.5000	7.8333	7	5.5000	5.5000	3	7.1666	7.6666	5.6666	5.3333	3.1666	4.1666	1
Total rank		10	12	8	6	6	2	9	11	7	5	3	4	1

**Table 4:** Assessment results of fixed-dimensional multimodal functions.

		GA	PSO	GSA	TLBO	GWO	WOA	TSA	MPA	RFO	RSA	AHA	HBA	SSVUBA
F_14_	avg	0.999359	2.175108	3.593904	2.265863	3.74346	3.108317	1.799941	0.998449	4.823742	5.383632	0.998004	1.592841	0.9980
	std	0.002474	2.938595	2.780694	1.150438	3.972512	3.536153	0.527866	0.000329	3.851995	3.816964	1.02 × 10^−16^	1.036634	0
	bsf	0.998702	0.998702	1.000208	0.99909	0.998702	0.998702	0.998599	0.997598	0.998004	2.156824	0.998004	0.998004	0.9980
	med	0.998716	0.998702	2.988748	2.276823	2.984193	0.998702	1.913947	0.998599	3.96825	2.98213	0.998004	0.998004	0.9980
	rank	4	7	10	8	11	9	6	3	12	13	2	5	1
F_15_	avg	0.005399	0.001685	0.002404	0.003172	0.006375	0.000664	0.000409	0.003939	0.005053	0.002185	0.00031	0.005509	0.0003
	std	0.008105	0.004936	0.001195	0.000394	0.009407	0.00035	7.6 × 10^−5^	0.005054	0.008991	0.001896	2.27 × 10^−8^	0.009072	2.3 × 10^−19^
	bsf	0.000776	0.000308	0.000805	0.002208	0.000308	0.000313	0.000265	0.00027	0.000307	0.000773	0.0003	0.000307	0.0003
	med	0.002075	0.000308	0.002312	0.003187	0.000308	0.000522	0.00039	0.002702	0.000653	0.001457	0.0003	0.000309	0.0003
	rank	11	5	7	8	13	4	3	9	10	6	2	12	1
F_16_	avg	−1.03058	−1.03060	−1.03060	−1.03060	−1.03060	−1.03060	−1.03056	−1.03056	−0.99082	−1.02581	−1.03162	−1.03162	−1.03163
	std	3.5 × 10^−5^	5.5 × 10^−16^	1.4 × 10^−16^	7.03 × 10^−15^	8.4 × 10^−9^	1.5 × 10^−10^	8.7 × 10^−6^	3.06 × 10^−5^	0.1825	0.011165	5.9 × 10^−13^	1.0 × 10^−16^	8.3 × 10^−17^
	bsf	−1.03060	−1.03060	−1.03060	−1.03060	−1.03060	−1.03060	−1.03058	−1.03057	−1.03163	−1.03159	−1.03163	−1.03163	−1.03163
	med	−1.03059	−1.03060	−1.03060	−1.03060	−1.03060	−1.03060	−1.03057	−1.03057	−1.03163	−1.03054	−1.03163	−1.03163	−1.03163
	rank	4	3	3	3	3	3	5	5	7	6	2	2	1
F_17_	avg	0.437274	0.785993	0.3978	0.3978	0.398166	0.398167	0.400369	0.399577	0.3978	0.439638	0.3978	0.3978	0.3978
	std	0.140844	0.72226	1.1 × 10^−16^	1.1 × 10^−16^	4.5 × 10^−7^	1.19 × 10^−6^	0.004484	0.003676	9.0 × 10^−16^	0.075523	7.1 × 10^−16^	6.4 × 10^−14^	4.0 × 10^−18^
	bsf	0.3978	0.3978	0.3978	0.3978	0.3978	0.3978	0.398331	0.397849	0.397887	0.398126	0.397887	0.397887	0.3978
	med	0.3978	0.3978	0.3978	0.3978	0.3978	0.3978	0.399331	0.398099	0.397887	0.411485	0.397887	0.397887	0.3978
	rank	6	8	1	1	2	3	5	4	1	7	1	1	1
F_18_	avg	4.36235	3.0020	3.0021	3.0000	3.002111	3.002109	3.0002	3.0021	13.8	7.423751	3	4.35	3.0000
	std	6.039455	2.5 × 10^−15^	1.8 × 10^−15^	6.3 × 10^−16^	1.0 × 10^−5^	1.56 × 10^−5^	0.0308	4.6 × 10^−16^	20.3563	19.78234	4.3 × 10^−16^	6.037384	0
	bsf	3.002101	3.0021	3.0021	3.0000	3.0021	3.0021	3.0001	3.0021	3	3.000011	3	3	3.0000
	med	3.003183	3.0021	3.0021	3.0021	3.002106	3.002102	3.00297	3.0021	3	3.000217	3	3	3.0000
	rank	8	3	4	1	6	5	2	4	10	9	1	7	1
F_19_	avg	−3.85049	−3.86278	−3.86278	−3.85752	−3.8583	−3.85682	−3.80279	−3.85884	−3.74604	−3.78545	−3.86278	−3.86081	−3.86278
	std	0.014825	1.6 × 10^−15^	1.5 × 10^−15^	0.00135	0.001695	0.002556	0.015203	2.2 × 10^−15^	0.282864	0.055424	2.3 × 10^−15^	0.003501	9.0 × 10^−16^
	bsf	−3.85892	−3.85892	−3.85892	−3.85864	−3.85892	−3.85892	−3.83276	−3.85884	−3.86278	−3.8432	−3.86278	−3.86278	−3.86278
	med	−3.85853	−3.85892	−3.85892	−3.85814	−3.8589	−3.8578	−3.80279	−3.85884	−3.86278	−3.79995	−3.86278	−3.86278	−3.86278
	rank	7	1	1	5	4	6	8	3	10	9	1	2	1
F_20_	avg	−2.82108	−3.25869	−3.322	−3.19797	−3.24913	−3.21976	−3.3162	−3.31777	−3.19517	−2.65147	−3.31011	−3.29793	−3.322
	std	0.385593	0.070568	0	0.031767	0.076495	0.090315	0.003082	8.34 × 10^−5^	0.311345	0.395844	0.036595	0.049393	0
	bsf	−3.31011	−3.31867	−3.322	−3.25848	−3.31867	−3.31866	−3.31788	−3.31797	−3.322	−3.05451	−3.322	−3.322	−3.322
	med	−2.96531	−3.31867	−3.322	−3.20439	−3.25921	−3.19197	−3.31728	−3.31778	−3.322	−2.79233	−3.322	−3.322	−3.322
	rank	11	6	1	9	7	8	3	2	10	12	4	5	1
F_21_	avg	−4.29971	−5.38381	−5.14352	−9.18098	−9.63559	−8.86747	−5.39669	−9.94449	−8.78928	−5.0552	−10.1532	−7.63362	−10.1532
	std	1.739082	3.016705	3.051569	0.120673	1.560428	2.26122	0.966938	0.532084	3.181731	3.2 × 10^−7^	1.06 × 10^−5^	3.97831	2.07 × 10^−7^
	bsf	−7.81998	−10.143	−10.143	−9.6542	−10.143	−10.1429	−7.49459	−10.143	−10.1532	−5.0552	−10.1532	−10.1532	−10.1532
	med	−4.15822	−5.09567	−3.64437	−9.14405	−10.1425	−10.1411	−5.49659	−10.143	−10.1524	−5.0552	−10.1532	−10.1532	−10.1532
	rank	12	9	10	4	3	5	8	2	6	11	1	7	1
F_22_	avg	−5.11231	−7.6247	−10.0746	−10.0386	−10.3921	−9.32799	−5.90758	−10.2757	−8.05397	−5.08767	−10.4029	−8.4968	−10.4029
	std	1.967685	3.538195	1.421736	0.397881	0.000176	2.177861	1.753184	0.245167	3.599306	7.2 × 10^−7^	0.00035	3.428023	1.61 × 10^−5^
	bsf	−9.10153	−10.3925	−10.3925	−10.3925	−10.3924	−10.3924	−9.05343	−10.3925	−10.4029	−5.08767	−10.4029	−10.4029	−10.4029
	med	−5.02463	−10.3925	−10.3925	−10.1734	−10.3921	−10.3908	−5.05743	−10.3925	−10.3962	−5.08767	−10.4029	−10.4029	−10.4029
	rank	11	9	4	5	2	6	10	3	8	12	1	7	1
F_23_	avg	−6.5556	−6.15864	−10.5364	−9.25502	−10.1201	−9.44285	−9.80005	−10.1307	−7.32853	−5.12847	−10.5334	−8.2629	−10.5364
	std	2.614706	3.731202	2.0 × 10^−15^	1.674862	1.812588	2.219704	1.604853	1.139028	4.034066	1.9 × 10^−6^	0.013601	3.580884	2.0 × 10^−15^
	bsf	−10.2124	−10.5259	−10.5364	−10.5235	−10.5258	−10.5257	−10.3579	−10.5259	−10.5364	−5.12848	−10.5364	−10.5364	−10.5364
	med	−6.55634	−4.50103	−10.5364	−9.66205	−10.5255	−10.5246	−10.3509	−10.5259	−10.508	−5.12847	−10.5364	−10.5364	−10.5364
	rank	10	11	1	7	4	6	5	3	9	12	2	8	1
Sum rank		84	62	42	51	55	55	55	38	83	97	17	56	10
Mean rank		8.4	6.2	4.2	5.1	5.5	5.5	5.5	3.8	8.3	9.7	1.7	5.6	1
Total rank		10	8	4	5	6	6	6	3	9	11	2	7	1

**Table 5:** *p*-values results from the Wilcoxon sum rank test.

Compared Algorithms	Test Function Type		
	Unimodal	High-Multimodal	Fixed-Multimodal
SSVUBA vs. HBA	6.5 × 10^−20^	7.58 × 10^−12^	3.91 × 10^−2^
SSVUBA vs. AHA	3.89 × 10^−13^	1.63 × 10^−11^	7.05 × 10^−7^
SSVUBA vs. RSA	1.79 × 10^−18^	1.63 × 10^−11^	1.44 × 10^−34^
SSVUBA vs. RFO	3.87 × 10^−23^	5.17 × 10^−12^	1.33 × 10^−7^
SSVUBA vs. MPA	1.01 × 10^−24^	4.02 × 10^−18^	1.39 × 10^−3^
SSVUBA vs. TSA	1.2 × 10^−22^	1.97 × 10^−21^	1.22 × 10^−25^
SSVUBA vs. WOA	9.7 × 10^−25^	1.89 × 10^−21^	9.11 × 10^−24^
SSVUBA vs. GWO	1.01 × 10^−24^	3.6 × 10^−16^	3.79 × 10^−20^
SSVUBA vs. TLBO	6.49 × 10^−23^	1.97 × 10^−21^	2.36 × 10^−25^
SSVUBA vs. GSA	1.97 × 10^−21^	1.97 × 10^−21^	5.2442 × 10^−2^
SSVUBA vs. PSO	1.01 × 10^−24^	1.97 × 10^−21^	3.71 × 10^−5^
SSVUBA vs. GA	1.01 × 10^−24^	1.97 × 10^−21^	1.44 × 10^−34^

**Table 6:** Results of sensitivity analysis of SSVUBA to *N*.

Objective Function	Number of Population Members			
	20	30	50	80
F_1_	3 × 10^−174^	3.9 × 10^−180^	5.02 × 10^−185^	1.6 × 10^−198^
F_2_	2.2 × 10^−92^	2.3 × 10^−95^	1.60 × 10^−99^	1.11 × 10^−107^
F_3_	4.3 × 10^−144^	1.9 × 10^−152^	2.01 × 10^−154^	1.3 × 10^−177^
F_4_	2.23 × 10^−60^	2.79 × 10^−62^	6.62 × 10^−59^	7.92 × 10^−67^
F_5_	0.022098	0.004318	2.54 × 10^−12^	9.24 × 10^−26^
F_6_	0	0	0	0
F_7_	0.000328	0.000181	9.00 × 10^−5^	2.99 × 10^−7^
F_8_	−12,569.5	−12,569.5	−12,569.4866	−12,569.5000
F_9_	0	0	0	0
F_10_	8.88 × 10^−16^	8.88 × 10^−16^	8.88 × 10^−16^	8.88 × 10^−16^
F_11_	0	0	0	0
F_12_	4.55 × 10^−23^	3.46 × 10^−29^	1.62 × 10^−32^	1.57 × 10^−32^
F_13_	1.54 × 10^−22^	1.88 × 10^−27^	7.65 × 10^−32^	1.35 × 10^−32^
F_14_	0.998	0.998	0.998	0.998
F_15_	0.000319	0.000314	0.000310	0.000308
F_16_	−1.03011	−1.03162	−1.03163	−1.03163
F_17_	0.399414	0.398137	0.3978	0.3978
F_18_	8.774656	3.000008	3	3
F_19_	−3.83542	−3.86173	−3.86278	−3.86278
F_20_	−2.83084	−2.99626	−3.322	−3.322
F_21_	−9.94958	−10.1532	−10.1532	−10.1532
F_22_	−10.4029	−10.4029	−10.4029	−10.4029
F_23_	−10.5358	−10.5364	−10.5364	−10.5364

**Table 7:** Results of sensitivity analysis of SSVUBA to *T*.

Objective Function	Maximum Number of Iterations			
	100	500	800	1000
F_1_	4.28 × 10^−19^	1.78 × 10^−93^	3.9 × 10^−149^	5.02 × 10^−185^
F_2_	4.2 × 10^−11^	4.15 × 10^−51^	4.98 × 10^−80^	1.60 × 10^−99^
F_3_	1.64 × 10^−11^	2.06 × 10^−76^	5.1 × 10^−127^	2.01 × 10^−154^
F_4_	4.07 × 10^−8^	3.7 × 10^−31^	3.49 × 10^−47^	6.62 × 10^−59^
F_5_	0.000271	1.25 × 10^−10^	1.6 × 10^−13^	2.54 × 10^−12^
F_6_	0	0	0	0
F_7_	0.0013	0.000162	9.62 × 10^−5^	9.00 × 10^−5^
F_8_	−12,569.5	−12,569.5	−12,569.5	−12,569.4866
F_9_	4.59 × 10^−9^	0	0	0
F_10_	2.89 × 10^−8^	8.88 × 10^−16^	8.88 × 10^−16^	8.88 × 10^−16^
F_11_	0	0	0	0
F_12_	2.31 × 10^−11^	2.18 × 10^−23^	1.47 × 10^−30^	1.62 × 10^−32^
F_13_	1.59 × 10^−10^	4.02 × 10^−23^	3.27 × 10^−29^	7.65 × 10^−32^
F_14_	0.998004	0.998004	0.998004	0.998
F_15_	0.000329	0.000312	0.000311	0.000310
F_16_	−1.0316	−1.03163	−1.03163	−1.03163
F_17_	0.397894	0.3978	0.3978	0.3978
F_18_	3.00398	3	3	3
F_19_	−3.86142	−3.86267	−3.86278	−3.86278
F_20_	−3.02449	−3.28998	−3.29608	−3.322
F_21_	−10.1516	−10.1532	−10.1532	−10.1532
F_22_	−10.4026	−10.4029	−10.4029	−10.4029
F_23_	−10.5362	−10.5364	−10.5364	−10.5364

**Table 8:** Results of sensitivity analysis of SSVUBA to the effectiveness of each case in Equation (4).

Objective Function	Maximum Number of Iterations		
	Mode 1	Mode 2	Mode 3
F_1_	1.63 × 10^−114^	2.80 × 10^−44^	5.02 × 10^−185^
F_2_	1.47 × 10^−59^	1.77 × 10^−22^	1.60 × 10^−99^
F_3_	4.72 × 10^−11^	5.70 × 10^−41^	2.01 × 10^−154^
F_4_	2.59 × 10^−36^	4.28 × 10^−23^	6.62 × 10^−59^
F_5_	28.77	1.58 × 10^−11^	2.54 × 10^−12^
F_6_	0	0	0
F_7_	0.000175	2.98 × 10^−4^	9.00 × 10^−5^
F_8_	−5593.8266	−12,569.4866	−12,569.4866
F_9_	0	0	0
F_10_	4.44 × 10^−18^	8.88 × 10^−16^	8.88 × 10^−16^
F_11_	0	0	0
F_12_	0.312707	1.15 × 10^−30^	1.62 × 10^−32^
F_13_	2.0409	1.84 × 10^−28^	7.65 × 10^−32^
F_14_	2.7155	0.998004	0.998
F_15_	0.00033149	0.001674	0.000310
F_16_	−1.03159	−0.35939	−1.03163
F_17_	0.39792	0.785468	0.3978
F_18_	3.653902	24.03998	3
F_19_	−3.84923	−3.38262	−3.86278
F_20_	−3.21768	−1.74165	−3.322
F_21_	−7.18942	−10.1532	−10.1532
F_22_	−7.63607	−10.4028	−10.4029
F_23_	−8.96944	−10.5363	−10.5364

**Table 9:** Assessment results of the CEC 2017 test functions.

		GA	PSO	GSA	TLBO	GWO	WOA	TSA	MPA	RFO	RSA	AHA	HBA	SSVUBA
C1	avg	9800	3960	296	19,800,000	325,000	8,470,000	296	3400	156	2470	2470	12,200	100
	std	6534	4906	302.5	4,466,000	117,700	25,410,000	302.5	4037	40,040	291.5	2431	28,380	526.9
	rank	7	6	3	11	9	10	4	5	2	4	5	8	1
C2	avg	5610	7060	7910	11,700	314	461	216	219	201	201	202	203	200
	std	4587	2409	2376	7007	7909	7766	839.3	738.1	81.95	104.17	507.1	897.6	11.44
	rank	9	10	11	12	7	8	5	6	2	3	3	4	1
C3	avg	8720	300	10,800	28,000	1540	23,400	10,800	300	301	1510	300	12,900	300
	std	6490	2.1 × 10^−10^	1782	9724	2079	4103	1760	0	52.69	27.94	2.64 × 10^−8^	5291	1.091 × 10^−10^
	rank	5	1	6	9	4	8	7	2	2	3	2	7	2
C4	avg	411	406	407	548	410	2390	407	406	403	404	404	478	400.03
	std	20.35	3.608	3.212	16.72	8.305	453.2	3.212	11.11	104.17	8.987	0.8701	21.45	0.0627
	rank	7	4	5	9	6	10	6	5	2	3	4	8	1
C5	avg	516	513	557	742	514	900	557	522	530	513	511	632	510.12
	std	7.623	7.194	9.24	38.83	6.71	87.45	9.251	11.55	64.13	26.73	4.037	38.5	4.3505
	rank	5	3	8	10	4	11	9	6	7	4	2	9	1
C6	avg	600	600	622	665	601	691	622	610	682	600	600	643	600
	std	0.07348	1.078	9.922	46.2	0.968	11.99	9.922	9.086	38.94	1.54	0.000165	18.15	0.0006776
	rank	1	2	4	6	2	8	5	3	7	2	2	5	2
C7	avg	728	719	715	1280	730	1860	715	741	713	713	721	878	723.32
	std	8.019	5.61	1.705	46.42	9.46	102.96	1.716	18.26	1.793	4.73	6.314	44.99	4.301
	rank	6	3	2	10	7	11	3	8	1	2	4	9	5
C8	avg	821	811	821	952	814	1070	821	823	829	809	810	917	809.42
	std	9.856	6.017	5.159	20.9	9.086	48.95	5.159	10.945	58.3	8.811	3.212	27.28	3.4342
	rank	6	4	7	10	5	11	7	7	8	1	3	9	2
C9	avg	910	900	900	6800	911	28,900	900	944	4670	910	900	2800	900
	std	16.72	6.5 × 10^−14^	6.5 × 10^−15^	1430	21.45	9614	0	115.5	2266	22	0.02497	921.8	0.01793
	rank	2	1	2	7	3	8	2	4	6	3	2	5	2
C10	avg	1720	1470	2690	5290	1530	7470	2690	1860	2590	1410	1420	4630	1437.42
	std	277.2	236.5	327.8	709.5	315.7	1496	327.8	324.5	455.4	38.5	288.2	677.6	155.188
	rank	6	4	9	11	5	12	10	7	8	1	2	10	3
C11	avg	1130	1110	1130	1270	1140	1920	1130	1180	1110	1110	1110	1200	1102.93
	std	26.18	6.908	11.55	43.78	59.51	2079	11.55	65.78	27.94	12.32	5.522	33.77	1.397
	rank	3	2	4	7	4	8	4	5	3	3	3	6	1
C12	avg	37,300	14,500	703,000	2.18 × 10^7^	625,000	1.84 × 10^8^	7.1 × 10^5^	1.98 × 10^6^	1630	15,200	10,300	620,000	1247.2
	std	38,280	12,430	46,310	2.31 × 10^7^	1.24 × 10^6^	1.87 × 10^9^	462,000	2.1 × 10^6^	217.8	2948	10,769	831,600	59.73
	rank	6	4	9	12	8	13	10	11	2	5	3	7	1
C13	avg	10,800	8600	11,100	415,000	9840	186,000,000	11,100	16,100	1320	6820	8020	12,900	1305.92
	std	9823	5632	2321	141,900	6193	150,700,000	2321	11,550	86.13	4686	7392	10,439	2.838
	rank	7	5	8	11	6	12	9	10	2	3	4	9	1
C14	avg	7050	1480	7150	412,000	3400	2,010,000	7150	1510	1450	1450	1460	25,510	1403.09
	std	8976	46.75	1639	250,800	2145	7,722,000	1639	56.21	61.6	24.64	35.75	32,780	4.466
	rank	7	4	8	10	6	11	9	5	2	3	3	9	1
C15	avg	9300	1710	18,000	47,500	3810	14,300,000	18,000	2240	1510	1580	1590	4490	1500.77
	std	9878	311.3	6050	16,500	4246	21,890,000	6050	628.1	18.04	140.8	52.8	3289	0.572
	rank	9	5	10	11	7	12	11	6	2	3	4	8	1
C16	avg	1790	1860	2150	3500	1730	3000	2150	1730	1820	1730	1650	2600	1604.82
	std	141.9	140.8	116.6	251.9	136.4	1320	116.6	139.7	253	132	55.99	322.3	1.089
	rank	4	6	7	10	3	9	8	4	5	4	2	8	1
C17	avg	1750	1760	1860	2630	1760	4340	1860	1770	1830	1730	1730	2170	1714.55
	std	43.78	52.25	118.8	209	34.43	348.7	118.8	37.62	193.6	37.95	19.91	232.1	10.384
	rank	3	4	7	9	5	10	8	5	6	2	3	8	1
C18	avg	15,700	14,600	8720	749,000	25,800	37,500,000	8720	23,400	1830	7440	12,500	194,000	1800.95
	std	14,080	13,090	5566	405,900	17,380	54,340,000	5566	15,400	14.85	4972	12,540	210,100	0.572
	rank	7	6	4	11	9	12	5	8	2	3	5	10	1
C19	avg	9690	2600	13,700	614,000	9870	2,340,000	45,000	2920	1920	1950	1950	5650	1900.9
	std	7447	2409	21,120	602,800	7007	17,820,000	20,900	2057	31.57	60.83	51.81	3443	0.495
	rank	7	4	9	11	8	12	10	5	2	3	4	6	1
C20	avg	2060	2090	2270	2870	2080	3790	2270	2090	2490	2020	2020	2440	2015.52
	std	66	68.53	89.87	224.4	57.2	486.2	89.87	54.23	267.3	27.83	24.53	206.8	10.637
	rank	3	5	6	9	4	10	7	6	8	2	3	7	1
C21	avg	2300	2280	2360	2580	2320	2580	2360	2250	2320	2230	2310	2400	2203.72
	std	48.18	59.4	31.02	67.87	7.7	202.4	31.02	66.44	74.58	47.85	23.1	69.19	22.385
	rank	5	4	8	10	7	11	9	3	8	2	6	9	1
C22	avg	2300	2310	2300	7180	2310	14,100	2300	2300	3530	2280	2300	2450	2283.76
	std	2.618	72.71	0.0792	1408	18.48	1133	0.077	12.98	932.8	14.63	20.24	910.8	41.91
	rank	3	4	4	7	5	8	4	4	6	1	4	5	2
C23	avg	2630	2620	2740	3120	2620	3810	2740	2620	2730	2610	2620	2820	2611.63
	std	14.74	10.153	43.01	91.41	9.317	240.9	43.01	9.559	267.3	4.532	6.083	55.99	4.323
	rank	4	3	6	8	4	9	7	4	5	1	4	7	2
C24	avg	2760	2690	2740	3330	2740	3480	2740	2730	2700	2620	2740	3010	2516.88
	std	16.39	118.8	6.072	178.2	9.603	240.9	6.105	70.84	80.74	87.56	7.59	46.97	42.229
	rank	7	3	6	9	7	10	7	5	4	2	7	8	1
C25	avg	2950	2920	2940	2910	2940	3910	2940	2920	2930	2920	2930	2890	2897.92
	std	21.23	27.5	16.94	19.36	25.96	280.5	16.83	26.29	22.99	13.86	21.78	15.29	0.539
	rank	7	4	6	3	7	8	7	5	5	5	6	1	2
C26	avg	3110	2950	34,400	7870	3220	7100	3440	2900	3460	3110	2970	4010	2849.81
	std	368.5	275	691.9	1001	469.7	3124	691.9	40.26	658.9	317.9	181.5	1017.5	105.919
	rank	5	3	12	11	6	10	7	2	8	6	4	9	1
C27	avg	3120	3120	3260	3410	3100	4810	3260	3090	3140	3110	3090	3200	3089.37
	std	21.12	27.5	45.87	90.31	23.98	675.4	45.87	3.058	23.54	22.99	2.464	0.0003399	0.506
	rank	5	6	8	9	3	10	9	2	6	4	3	7	1
C28	avg	3320	3320	3460	3400	3390	5090	3460	3210	3400	2300	3300	3260	3100
	std	138.6	134.2	37.18	130.9	112.2	346.5	37.18	124.3	144.1	136.4	147.4	46.86	0.00006974
	rank	6	7	9	8	7	10	10	3	9	1	5	4	2
C29	avg	3250	3200	3450	4560	3190	8890	3450	3210	3210	3210	3170	3620	3146.26
	std	90.2	57.53	188.1	543.4	47.19	1562	188.1	56.87	121	62.26	27.17	222.2	14.08
	rank	6	4	7	9	3	10	8	5	6	6	2	8	1
C30	avg	537,000	351,000	1,300,000	4,030,000	298,000	18,800,000	940,000	421,000	305,000	296,000	297,000	6490	3414.92
	std	700,700	555,500	400,400	1,760,000	580,800	146,300,000	396,000	624,800	489,500	23,540	504,900	8844	29.491
	rank	9	7	11	12	5	13	10	8	6	3	4	2	1
Sum rank		167	128	206	282	166	305	217	159	142	88	108	212	44
Mean rank		5.5666	4.2666	6.8666	9.4	5.5333	10.1666	7.2333	5.3	4.7333	2.9333	3.6	7.0666	1.4666
Total rank		8	4	9	12	7	13	11	6	5	2	3	10	1

**Table 10:** Performance of optimization algorithms in the pressure vessel design problem.

Algorithm	Optimum Variables				Optimum Cost
	*T_s_*	*T_h_*	*R*	*L*	
SSVUBA	0.7789938	0.3850896	40.3607	199.3274	5884.8824
AHA	0.778171	0.384653	40.319674	199.999262	5885.5369
RSA	0.8400693	0.4189594	43.38117	161.5556	6034.7591
RFO	0.81425	0.44521	42.20231	176.62145	6113.3195
MPA	0.787576	0.389521	40.80024	200.0000	5916.780
TSA	0.788411	0.389289	40.81314	200.0000	5920.592
WOA	0.818188	0.440563	42.39296	177.8755	5922.621
GWO	0.855898	0.423602	44.3436	158.2636	6043.384
TLBO	0.827417	0.422962	42.25185	185.782	6169.909
GSA	1.098868	0.961043	49.9391	171.5271	11611.53
PSO	0.761417	0.404349	40.93936	200.3856	5921.556
GA	1.112756	0.91749	44.99143	181.8211	6584.748

**Table 11:** Statistical results of optimization algorithms for the pressure vessel design problem.

Algorithm	Best	Mean	Worst	Std. Dev.	Median
SSVUBA	5884.8824	5888.170	5895.379	23.716394	5887.907
AHA	5885.5369	5885.53823	5885.85190	31.1378	5888.406
RSA	6034.7591	6042.051	6045.914	31.204538	6040.142
RFO	6113.3195	6121.207	6132.519	38.26314	6119.021
MPA	5916.780	5892.155	5897.036	28.95315	5890.938
TSA	5920.592	5896.238	5899.34	13.92114	5895.363
WOA	5922.621	6069.87	7400.504	66.6719	6421.248
GWO	6043.384	6482.488	7256.718	327.2687	6402.599
TLBO	6169.909	6331.823	6517.565	126.7103	6323.373
GSA	11611.53	6846.016	7165.019	5795.258	6843.104
PSO	5921.556	6269.017	7011.356	496.525	6117.581
GA	6584.748	6649.303	8011.845	658.0492	7592.079

**Table 12:** Performance of optimization algorithms in the speed reducer design problem.

Algorithm				Optimum Variables				Optimum Cost
	*b*	*m*	*p*	*l*_1_	*l*_2_	*d*_1_	*d*_2_	
SSVUBA	3.50003	0.700007	17	7.3	7.8	3.350210	5.286681	2996.3904
HBA	3.4976	0.7	17	7.3000	7.8000	3.3501	5.2857	2996.4736
AHA	3.50000	0.7	17	7.300001	7.7153201	3.350212	5.286655	2996.4711
RSA	3.50279	0.7	17	7.30812	7.74715	3.35067	5.28675	2996.5157
RFO	3.509368	0.7	17	7.396137	7.800163	3.359927	5.289782	3005.1373
MPA	3.503621	0.7	17	7.300511	7.8	3.353181	5.291754	3001.85
TSA	3.508724	0.7	17	7.381576	7.815781	3.359761	5.289781	3004.591
WOA	3.502049	0.7	17	8.300581	7.800055	3.354323	5.289728	3009.07
GWO	3.510537	0.7	17	7.410755	7.816089	3.359987	5.28979	3006.232
TLBO	3.51079	0.7	17	7.300001	7.8	3.462993	5.292228	3033.897
GSA	3.602088	0.7	17	8.300581	7.8	3.371579	5.292239	3054.478
PSO	3.512289	0.7	17	8.350585	7.8	3.364117	5.290737	3070.936
GA	3.522166	0.7	17	8.370586	7.8	3.368889	5.291733	3032.335

**Table 13:** Statistical results of optimization algorithms for the speed reducer design problem.

Algorithm	Best	Mean	Worst	Std. Dev.	Median
SSVUBA	2996.3904	3000.0294	3001.627	1.6237192	2999.0614
HBA	2996.4736	3001.279	30002.716	4.163725	3000.7196
AHA	2996.4711	3000.471	3002.473	2.015234	3000.1362
RSA	2996.5157	3002.164	3007.394	5.219620	3000.7315
RFO	3005.1373	3012.031	3027.619	10.36912	3010.641
MPA	3001.85	3003.841	3008.096	1.934636	3003.387
TSA	3004.591	3010.055	3012.966	5.846116	3008.727
WOA	3009.07	3109.601	3215.671	79.74963	3109.601
GWO	3006.232	3033.083	3065.245	13.03683	3031.271
TLBO	3033.897	3070.211	3109.127	18.09951	3069.902
GSA	3054.478	3174.774	3368.584	92.70225	3161.173
PSO	3070.936	3190.985	3317.84	17.14257	3202.666
GA	3032.335	3299.944	3624.534	57.10336	3293.263

**Table 14:** Performance of optimization algorithms in the welded beam design problem.

Algorithm		Optimum Variables			Optimum Cost
	*h*	*l*	*t*	*b*	
SSVUBA	0.205730	3.4705162	9.0366314	0.2057314	1.724852
HBA	0.2057	3.4704	9.0366	0.2057	1.72491
AHA	0.205730	3.470492	9.036624	0.205730	1.724853
RSA	0.14468	3.514	8.9251	0.21162	1.6726
RFO	0.21846	3.51024	8.87254	0.22491	1.86612
MPA	0.205563	3.474846	9.035799	0.205811	1.727656
TSA	0.205678	3.475403	9.036963	0.206229	1.728992
WOA	0.197411	3.315061	9.998	0.201395	1.8225
GWO	0.205611	3.472102	9.040931	0.205709	1.727467
TLBO	0.204695	3.536291	9.00429	0.210025	1.761207
GSA	0.147098	5.490744	10.0000	0.217725	2.175371
PSO	0.164171	4.032541	10.0000	0.223647	1.876138
GA	0.206487	3.635872	10.0000	0.203249	1.838373

**Table 15:** Statistical results of optimization algorithms for the welded beam design problem.

Algorithm	Best	Mean	Worst	Std. Dev.	Median
SSVUBA	1.724852	1.726331	1.72842	0.004328	1.725606
HBA	1.72491	1.72685	1.72485	0.007132	1.725854
AHA	1.724853	1.727123	1.7275528	0.005123	1.725824
RSA	1.6726	1.703415	1.762140	0.017425	1.726418
RFO	1.86612	1.892058	2.016378	0.007960	1.88354
MPA	1.727656	1.728861	1.729097	0.000287	1.72882
TSA	1.728992	1.730163	1.730599	0.001159	1.730122
WOA	1.8225	2.234228	3.053587	0.325096	2.248607
GWO	1.727467	1.732719	1.744711	0.004875	1.730455
TLBO	1.761207	1.82085	1.8767	0.027591	1.823326
GSA	2.175371	2.548709	3.008934	0.256309	2.499498
PSO	1.876138	2.122963	2.324201	0.034881	2.100733
GA	1.838373	1.365923	2.038823	0.13973	1.939149

**Table 16:** Performance of optimization algorithms for the tension/compression spring design problem.

Algorithm		Optimum Variables		Optimum Cost
	*d*	*D*	*p*	
SSVUBA	0.051704	0.357077	11.26939	0.012665
HBA	0.0506	0.3552	11.373	0.012707
AHA	0.051897	0.361748	10.689283	0.012666
RSA	0.057814	0.58478	4.0167	0.01276
RFO	0.05189	0.36142	11.58436	0.01321
MPA	0.050642	0.340382	11.97694	0.012778
TSA	0.049686	0.338193	11.95514	0.012782
WOA	0.04951	0.307371	14.85297	0.013301
GWO	0.04951	0.312859	14.08679	0.012922
TLBO	0.050282	0.331498	12.59798	0.012814
GSA	0.04951	0.314201	14.0892	0.012979
PSO	0.049609	0.307071	13.86277	0.013143
GA	0.049757	0.31325	15.09022	0.012881

**Table 17:** Statistical results of optimization algorithms for the tension/compression spring design problem.

Algorithm	Best	Mean	Worst	Std. Dev.	Median
SSVUBA	0.012665	0.012687	0.012696	0.001022	0.012684
HBA	0.012707	0.0127162	0.0128012	0.006147	0.012712
AHA	0.012666	0.0126976	0.0127271	0.001566	0.012692
RSA	0.01276	0.012792	0.012804	0.007413	0.012782
RFO	0.01321	0.01389	0.015821	0.006137	0.013768
MPA	0.012778	0.012795	0.012826	0.005668	0.012798
TSA	0.012782	0.012808	0.012832	0.00419	0.012811
WOA	0.013301	0.014947	0.018018	0.002292	0.013308
GWO	0.012922	0.01459	0.017995	0.001636	0.014143
TLBO	0.012814	0.012952	0.013112	0.007826	0.012957
GSA	0.012979	0.013556	0.014336	0.000289	0.013484
PSO	0.013143	0.014158	0.016393	0.002091	0.013115
GA	0.012881	0.013184	0.015347	0.000378	0.013065

## Data Availability

Not applicable.

## Appendix A

The information of the objective functions utilized in the simulation section is shown in Table A1, Table A2 and Table A3.

**Table A1:** Information of unimodal functions.

Objective Function	Range	Dimensions	$F_{min}$
$F_{1}\left(x\right)=\sum_{i=1}^{m}x_{i}^{2}$	[−100, 100]	30	0
$F_{2}\left(x\right)=\sum_{i=1}^{m}\left|x_{i}\right|+\prod_{i=1}^{m}\left|x_{i}\right|$	[−10, 10]	30	0
$F_{3}\left(x\right)=\sum_{i=1}^{m}{\left(\sum_{j=1}^{i}x_{i}\right)}^{2}$	[−100, 100]	30	0
$F_{4}\left(x\right)=max\left\{\left|x_{i}\right|\;,\;\;1\leq i\leq m\;\right\}$	[−100, 100]	30	0
$F_{5}\left(x\right)=\sum_{i=1}^{m-1}\left[100{\left(x_{i+1}-x_{i}^{2}\right)}^{2}+{\left(x_{i}-1\right)}^{2})\right]$	[−30, 30]	30	0
$F_{6}\left(x\right)=\sum_{i=1}^{m}{\left(\left[x_{i}+0.5\right]\right)}^{2}$	[−100, 100]	30	0
$F_{7}\left(x\right)=\sum_{i=1}^{m}ix_{i}^{4}+random\left(0,1\right)$	[−1.28, 1.28]	30	0

**Table A2:** Information of high-dimensional multimodal functions.

Objective Function	Range	Dimensions	$F_{min}$
$F_{8}\left(x\right)=\sum_{i=1}^{m}-x_{i}\;\sin\left(\sqrt{\left|x_{i}\right|}\right)$	[−500, 500]	30	−12,569
$F_{9}\left(x\right)=\sum_{i=1}^{m}\left[\;x_{i}^{2}-10\cos\left(2\pi x_{i}\right)+10\right]$	[−5.12, 5.12]	30	0
$F_{10}\left(x\right)=-20\exp\left(-0.2\sqrt{\frac{1}{m}\sum_{i=1}^{m}x_{i}^{2}}\right)-\exp\left(\frac{1}{m}\sum_{i=1}^{m}\cos\left(2\pi x_{i}\right)\right)+20+e$	[−32, 32]	30	0
$F_{11}\left(x\right)=\frac{1}{4000}\sum_{i=1}^{m}x_{i}^{2}-\prod_{i=1}^{m}cos\left(\frac{x_{i}}{\sqrt{i}}\right)+1$	[−600, 600]	30	0
$\begin{array}{cc} F_{12}\left(x\right)=\frac{\pi }{m}\;\{10 & \sin\left(\pi y_{1}\right)\\ & +\sum_{i=1}^{m}{\left(y_{i}-1\right)}^{2}\left[1+10\sin^{2}\left(\pi y_{i+1}\right)\right]+{\left(y_{n}-1\right)}^{2}\}\\ & +\sum_{i=1}^{m}u\left(x_{i},10,100,4\right) \end{array}$ $u\left(x_{i},a,i,n\right)=\{\begin{array}{c} k{\left(x_{i}-a\right)}^{n}\;\;\;\;\;\;\;\;\;\;\;\;\;\;\;x_{i}>-a\\ 0\;\;\;\;\;\;\;\;\;\;\;\;\;\;\;\;\;\;\;-a<x_{i}<a\\ k{\left(-x_{i}-a\right)}^{n}\;\;\;\;\;\;\;\;\;\;\;x_{i}<-a \end{array}$	[−50, 50]	30	0
$\begin{array}{cc} F_{13}\left(x\right)=0.1\{\;\sin & {}^{2}\left(3\pi x_{1}\right)\\ & +\sum_{i=1}^{m}{\left(x_{i}-1\right)}^{2}\left[1+\sin^{2}\left(3\pi x_{i}+1\right)\right]+{\left(x_{n}-1\right)}^{2}[1\\ & +\sin^{2}\left(2\pi x_{m}\right)]\}+\sum_{i=1}^{m}u\left(x_{i},5,100,4\right) \end{array}$	[−50, 50]	30	0

**Table A3:** Information of fixed-dimensional multimodal functions.

Objective Function	Range	Dimensions	$F_{min}$
$F_{14}\left(x\right)={\left(\frac{1}{500}+\sum_{j=1}^{25}\frac{1}{j+\sum_{i=1}^{2}{\left(x_{i}-a_{ij}\right)}^{6}}\right)}^{-1}$	[−65.53, 65.53]	2	0.998
$F_{15}\left(x\right)=\sum_{i=1}^{11}{\left[a_{i}-\frac{x_{1}\left(b_{i}^{2}+b_{i}x_{2}\right)}{b_{i}^{2}+b_{i}x_{3}+x_{4}}\right]}^{2}$	[−5, 5]	4	0.00030
$F_{16}\left(x\right)=4x_{1}^{2}-2.1x_{1}^{4}+\frac{1}{3}x_{1}^{6}+x_{1}x_{2}-4x_{2}^{2}+4x_{2}^{4}$	[−5, 5]	2	−1.0316
$F_{17}\left(x\right)={\left(x_{2}-\frac{5.1}{4\pi^{2}}x_{1}^{2}+\frac{5}{\pi }x_{1}-6\right)}^{2}+10\left(1-\frac{1}{8\pi }\right)\cos x_{1}+10$	[−5, 10] × [0, 15]	2	0.398
$\begin{array}{cc} F_{18}\left(x\right)=[1+(x_{1} & {+x_{2}+1)}^{2}(19-14x_{1}+3x_{1}^{2}-14x_{2}+6x_{1}x_{2}\\ & +3x_{2}^{2})]\times [30+{\left(2x_{1}-3x_{2}\right)}^{2}\times (18-32x_{1}+12x_{1}^{2}\\ & +48x_{2}-36x_{1}x_{2}+27x_{2}^{2})] \end{array}$	[−5, 5]	2	3
$F_{19}\left(x\right)=-\sum_{i=1}^{4}c_{i}\exp\left(-\sum_{j=1}^{3}a_{ij}{\left(x_{j}-P_{ij}\right)}^{2}\right)$	[0, 1]	3	−3.86
$F_{20}\left(x\right)=-\sum_{i=1}^{4}c_{i}\exp\left(-\sum_{j=1}^{6}a_{ij}{\left(x_{j}-P_{ij}\right)}^{2}\right)$	[0, 1]	6	−3.22
$F_{21}\left(x\right)=-\sum_{i=1}^{5}{\left[\left(X-a_{i}\right){\left(X-a_{i}\right)}^{T}+6c_{i}\right]}^{-1}$	[0, 10]	4	−10.1532
$F_{22}\left(x\right)=-\sum_{i=1}^{7}{\left[\left(X-a_{i}\right){\left(X-a_{i}\right)}^{T}+6c_{i}\right]}^{-1}$	[0, 10]	4	−10.4029
$F_{23}\left(x\right)=-\sum_{i=1}^{10}{\left[\left(X-a_{i}\right){\left(X-a_{i}\right)}^{T}+6c_{i}\right]}^{-1}$	[0, 10]	4	−10.5364

**Table A4:** Information of CEC 2017 test functions.

		Functions	$f_{min}$
Unimodal functions	C1	Shifted and Rotated Bent Cigar Function	100
	C2	Shifted and Rotated Sum of Different Power Function	200
	C3	Shifted and Rotated Zakharov Function	300
Simple multimodal functions	C4	Shifted and Rotated Rosenbrock Function	400
	C5	Shifted and Rotated Rastrigin Function	500
	C6	Shifted and Rotated Expanded Scaffer Function	600
	C7	Shifted and Rotated Lunacek Bi_Rastrigin Function	700
	C8	Shifted and Rotated Non-Continuous Rastrigin Function	800
	C9	Shifted and Rotated Levy Function	900
	C10	Shifted and Rotated Schwefel Function	1000
Hybrid functions	C11	Hybrid Function 1 (*N* = 3)	1100
	C12	Hybrid Function 2 (*N* = 3)	1200
	C13	Hybrid Function 3 (*N* = 3)	1300
	C14	Hybrid Function 4 (*N* = 4)	1400
	C15	Hybrid Function 5 (*N* = 4)	1500
	C16	Hybrid Function 6 (*N* = 4)	1600
	C17	Hybrid Function 6 (*N* = 5)	1700
	C18	Hybrid Function 6 (*N* = 5)	1800
	C19	Hybrid Function 6 (*N* = 5)	1900
	C20	Hybrid Function 6 (*N* = 6)	2000
Composition functions	C21	Composition Function 1 (*N* = 3)	2100
	C22	Composition Function 2 (*N* = 3)	2200
	C23	Composition Function 3 (*N* = 4)	2300
	C24	Composition Function 4 (*N* = 4)	2400
	C25	Composition Function 5 (*N* = 5)	2500
	C26	Composition Function 6 (*N* = 5)	2600
	C27	Composition Function 7 (*N* = 6)	2700
	C28	Composition Function 8 (*N* = 6)	2800
	C29	Composition Function 9 (*N* = 3)	2900
	C30	Composition Function 10 (*N* = 3)	3000

## References

[1] Dhiman G. (2021). SSC: A hybrid nature-inspired meta-heuristic optimization algorithm for engineering applications. Knowl.-Based Syst..

[2] Fletcher R. (2013). Practical Methods of Optimization.

[3] Cavazzuti M. (2013). Deterministic Optimization. Optimization Methods: From Theory to Design Scientific and Technological Aspects in Mechanics.

[4] Dehghani M., Montazeri Z., Dehghani A., Samet H., Sotelo C., Sotelo D., Ehsanifar A., Malik O.P., Guerrero J.M., Dhiman G. (2020). DM: Dehghani Method for modifying optimization algorithms. Appl. Sci..

[5] Iba K. (1994). Reactive power optimization by genetic algorithm. IEEE Trans. Power Syst..

[6] Banerjee A., De S.K., Majumder K., Das V., Giri D., Shaw R.N., Ghosh A. (2022). Construction of effective wireless sensor network for smart communication using modified ant colony optimization technique. Advanced Computing and Intelligent Technologies.

[7] Zhang X., Dahu W. (2019). Application of artificial intelligence algorithms in image processing. J. Vis. Commun. Image Represent..

[8] Djenouri Y., Belhadi A., Belkebir R. (2018). Bees swarm optimization guided by data mining techniques for document information retrieval. Expert Syst. Appl..

[9] Chaudhuri A., Sahu T.P. (2021). Feature selection using Binary Crow Search Algorithm with time varying flight length. Expert Syst. Appl..

[10] Singh T., Saxena N., Khurana M., Singh D., Abdalla M., Alshazly H. (2021). Data Clustering Using Moth-Flame Optimization Algorithm. Sensors.

[11] Fathy A., Alharbi A.G., Alshammari S., Hasanien H.M. (2022). Archimedes optimization algorithm based maximum power point tracker for wind energy generation system. Ain Shams Eng. J..

[12] Hasan M.Z., Al-Rizzo H. (2020). Beamforming optimization in internet of things applications using robust swarm algorithm in conjunction with connectable and collaborative sensors. Sensors.

[13] Wolpert D.H., Macready W.G. (1997). No free lunch theorems for optimization. IEEE Trans. Evol. Comput..

[14] Trojovský P., Dehghani M. (2022). Pelican Optimization Algorithm: A Novel Nature-Inspired Algorithm for Engineering Applications. Sensors.

[15] Dehghani M., Trojovský P. (2021). Teamwork Optimization Algorithm: A New Optimization Approach for Function Minimization/Maximization. Sensors.

[16] Goldberg D.E., Holland J.H. (1988). Genetic Algorithms and Machine Learning. Mach. Learn..

[17] Dorigo M., Maniezzo V., Colorni A. (1996). Ant system: Optimization by a colony of cooperating agents. IEEE Trans. Syst. Man Cybern. Part B (Cybern.).

[18] Kennedy J., Eberhart R. Particle Swarm Optimization. Proceedings of the ICNN’95—International Conference on Neural Networks.

[19] Kirkpatrick S., Gelatt C.D., Vecchi M.P. (1983). Optimization by simulated annealing. Science.

[20] Yang X.-S. (2010). Firefly algorithm, stochastic test functions and design optimisation. Int. J. Bio-Inspir. Comput..

[21] Rao R.V., Savsani V.J., Vakharia D. (2011). Teaching–learning-based optimization: A novel method for constrained mechanical design optimization problems. Comput.-Aided Des..

[22] Geem Z.W., Kim J.H., Loganathan G.V. (2001). A new heuristic optimization algorithm: Harmony search. Simulation.

[23] Li X.-l. (2002). An optimizing method based on autonomous animats: Fish-swarm algorithm. Syst. Eng.-Theory Pract..

[24] Mirjalili S., Mirjalili S.M., Lewis A. (2014). Grey wolf optimizer. Adv. Eng. Softw..

[25] Rashedi E., Nezamabadi-Pour H., Saryazdi S. (2009). GSA: A gravitational search algorithm. Inf. Sci..

[26] Mirjalili S., Lewis A. (2016). The whale optimization algorithm. Adv. Eng. Softw..

[27] Faramarzi A., Heidarinejad M., Mirjalili S., Gandomi A.H. (2020). Marine Predators Algorithm: A nature-inspired metaheuristic. Expert Syst. Appl..

[28] Kaur S., Awasthi L.K., Sangal A.L., Dhiman G. (2020). Tunicate Swarm Algorithm: A new bio-inspired based metaheuristic paradigm for global optimization. Eng. Appl. Artif. Intell..

[29] Zamani H., Nadimi-Shahraki M.H., Gandomi A.H. (2021). QANA: Quantum-based avian navigation optimizer algorithm. Eng. Appl. Artif. Intell..

[30] Zamani H., Nadimi-Shahraki M.H., Gandomi A.H. (2019). CCSA: Conscious neighborhood-based crow search algorithm for solving global optimization problems. Appl. Soft Comput..

[31] Hayyolalam V., Kazem A.A.P. (2020). Black widow optimization algorithm: A novel meta-heuristic approach for solving engineering optimization problems. Eng. Appl. Artif. Intell..

[32] Połap D., Woźniak M. (2021). Red fox optimization algorithm. Expert Syst. Appl..

[33] Zhao W., Wang L., Mirjalili S. (2022). Artificial hummingbird algorithm: A new bio-inspired optimizer with its engineering applications. Comput. Methods Appl. Mech. Eng..

[34] Abualigah L., Abd Elaziz M., Sumari P., Geem Z.W., Gandomi A.H. (2022). Reptile Search Algorithm (RSA): A nature-inspired meta-heuristic optimizer. Expert Syst. Appl..

[35] Hashim F.A., Houssein E.H., Hussain K., Mabrouk M.S., Al-Atabany W. (2022). Honey Badger Algorithm: New metaheuristic algorithm for solving optimization problems. Math. Comput. Simul..

[36] Zamani H., Nadimi-Shahraki M.H., Gandomi A.H. (2022). Starling murmuration optimizer: A novel bio-inspired algorithm for global and engineering optimization. Comput. Methods Appl. Mech. Eng..

[37] Yao X., Liu Y., Lin G. (1999). Evolutionary programming made faster. IEEE Trans. Evol. Comput..

[38] Awad N., Ali M., Liang J., Qu B., Suganthan P. (2016). Problem Definitions Evaluation Criteria for the CEC 2017 Special Session and Competition on Single Objective Real-Parameter Numerical Optimization.

[39] Wilcoxon F. (1992). Individual comparisons by ranking methods. Breakthroughs in Statistics.

[40] Nadimi-Shahraki M.H., Fatahi A., Zamani H., Mirjalili S., Abualigah L., Abd Elaziz M. (2021). Migration-based moth-flame optimization algorithm. Processes.

[41] Kannan B., Kramer S.N. (1994). An augmented Lagrange multiplier based method for mixed integer discrete continuous optimization and its applications to mechanical design. J. Mech. Des..

[42] Gandomi A.H., Yang X.-S. (2011). Benchmark problems in structural optimization. Computational Optimization, Methods and Algorithms.

[43] Mezura-Montes E., Coello C.A.C. (2005). Useful infeasible solutions in engineering optimization with evolutionary algorithms. Proceedings of the Mexican International Conference on Artificial Intelligence.

